# Phase Diagram-Enabled Curcumin Isolation and Purification by Utilizing Resorcinol as Cocrystal Former and Additive

**DOI:** 10.1021/acsomega.5c12529

**Published:** 2026-03-17

**Authors:** Yu-Rong Weng, Ya-Hsuan Huang, Jhe-Wei Wu, Dhanang Edy Pratama, Tu Lee

**Affiliations:** Department of Chemical and Materials Engineering, National Central University, 300 Zhongda Road, Zhongli District, Taoyuan City 320317, Taiwan, R. O. C.

## Abstract

In this study, cocrystallization was explored as a method for purifying a compound from a complex mixture. Using the case of curcumin purification, resorcinol was selected as the additional component due to its propensity to form a cocrystal with curcumin in a mole ratio of 1:1. To enable a proper process design, a ternary phase diagram of curcumin-resorcinol-ethanol at 25 °C was constructed. However, it was found that resorcinol can only be suitably used if the curcumin content in the crude powders is at least 70 wt %, otherwise complete solubilization would occur. By taking this into account, starting from raw turmeric powders, the following operations were carried out: (1) extraction, (2) solid–liquid equilibration, and (3) two stages of cocrystal formation-dissociation. Finally, 96.06 ± 1.19 wt % curcumin purity with an overall yield of 0.26 ± 0.02 wt % was obtained. Furthermore, coformer recycling was carried out by antisolvent addition and evaporative crystallization of the mother liquor, with a recovery yield of up to 80.6 ± 1.40 wt %. Interestingly, the coformer could also be used directly as an additive to the curcumin-ethanol suspension, thereby entirely skipping the cocrystal formation-dissociation steps. Starting from raw material with 70 wt % curcumin purity, this method could produce a superior curcumin purity of 85.3 ± 2.0 wt % against 78.5 ± 1.6 wt % without any resorcinol addition, both at similar yield values.

## Graphical abstract

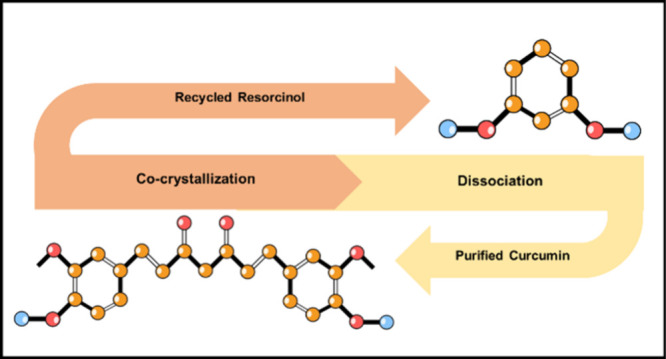

## Introduction

Curcumin is the principal curcuminoid found in turmeric. Turmeric extract consists of volatile essential oils and a heavy yellow-brown fraction, with the characteristic yellow color mainly attributed to three curcuminoids: curcumin (CUR), demethoxycurcumin (DMC), and bisdemethoxycurcumin (BDMC), as shown in Figure 1.^1^ The total curcuminoid content in turmeric typically ranges from 2 to 9 wt %,^2^ with CUR being the dominant bioactive component, ranging from 0.58 to 3.14 wt %.^3^ Besides that, the crude extracts of curcumin contain a variety of other components, such as essential oils and other small molecules.

**Figure 1:**
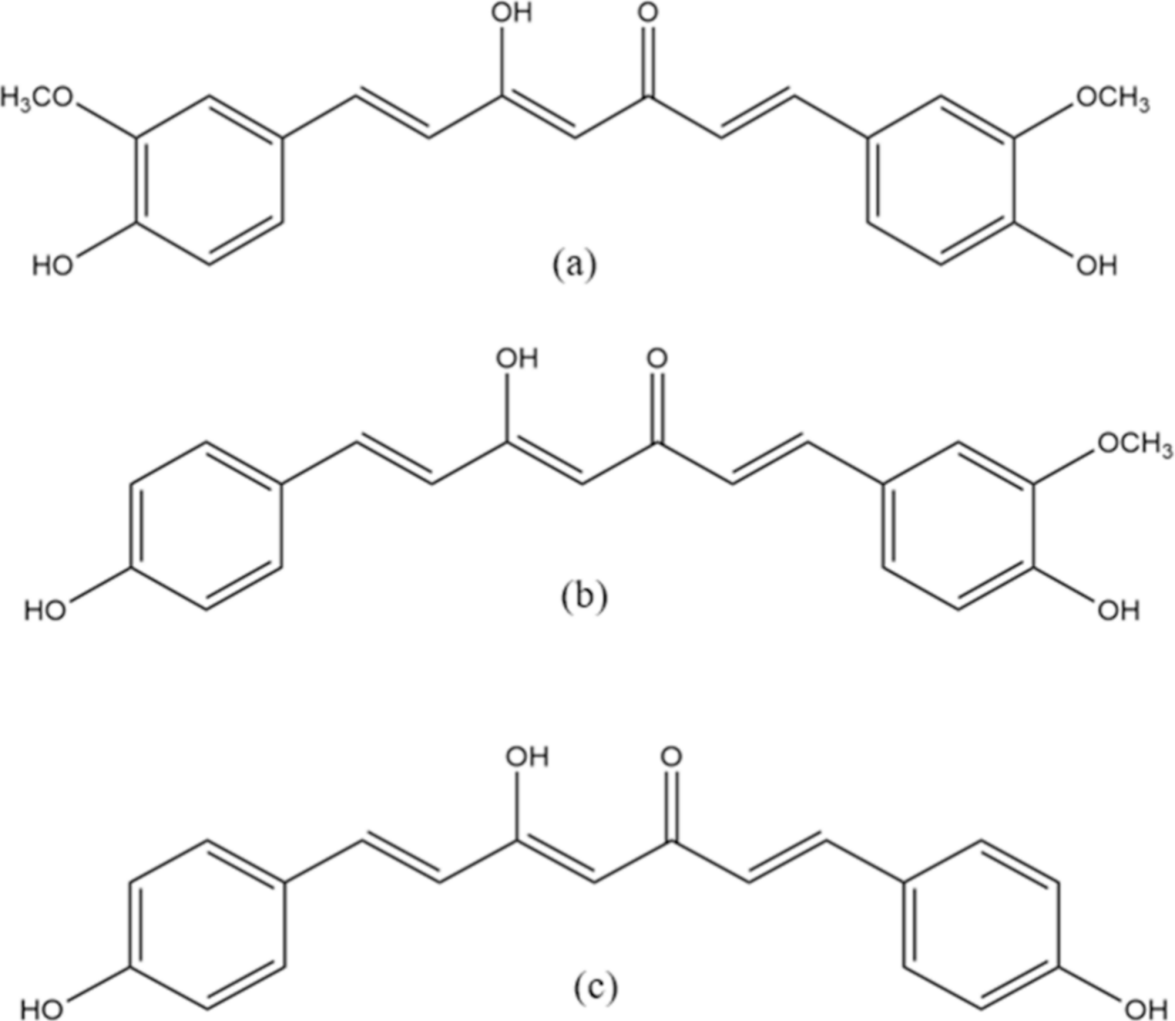
Chemical structures of curcuminoids: (a) CUR, (b) DMC, and (c) BDMC.

### Existing Purification Technique of Curcumin

To obtain high-purity curcumin from these mixtures, various purification techniques are available, including conventional methods such as column chromatography and crystallization, as well as advanced techniques such as high-speed countercurrent chromatography and supercritical fluid chromatography, as summarized in Table 1. In addition, novel solvents such as deep eutectic solvents or ionic liquids have demonstrated significant advantages in terms of solubility and selectivity for either chemical synthesis or extraction.^4,5^ In terms of the extraction of natural product isolation, these solvents are commonly used for the extraction step. However, it should be noted that both ionic liquid and deep eutectic solvent are known to have a high vapor pressure,^6,7^ which may complicate their removal from the final solid product and compromise the final purity due to the difficulty in drying.

**Table 1:** Some Examples of Curcumin Purification Studies

**CUR source and initial content**	**mode**	**CUR purity (wt %)**	**yield (%)**	**ref**
crude extract in acetone (containing 22.8% CUR)	silica gel column chromatography with chloroform/methanol as mobile phase, followed by recrystallization in chloroform/methanol/petroleum ether	qualitatively pure by HPLC	not stated	^8^
crude CUR powders (containing 78.6% CUR)	three-stage cooling crystallization from 60° to 20 °C in isopropanol under various seeding parameters	up to 98.99% of pure Form I CUR at the third stage	up to 50% at the third stage^a^	^9^
extract solution from turmeric powder	high-speed counter-current chromatography (HSCCC) in two-phase solvent system: hexane/chloroform/methanol/water (5/10/7.5/2.5, v/v)	>98	4.4^b^	^10^
methanolic extract of curcumin	supercritical fluid chromatography in Viridis BEH OBD column using supercritical fluid CO_2_ containing 8 to 15% methanol (containing 10 mM oxalic acid) at 80 mL/min	97.9	20.8^c^	^11^
crude curcumin (67–75%)	single-stage seeded cooling crystallization in various solvents/cosolvents with/without antisolvent	90.1–99.4 (without antisolvent)	13–62^d^ (without antisolvent)	^12^
		85.5–96.2 (with antisolvent)	36–79^d^ (with antisolvent)	
crude curcumin (79.6%)	single-stage cocrystallization by using RES as the coformer in toluene/ethyl acetate 90/10 (v/v) and ethanol	83.6–86.4 (in toluene/ethyl acetate 90/10 (v/v))	86.3–96.4 (in toluene/ethyl acetate 90/10 (v/v))^d^	^13^
		83.5 (in ethanol)	78.4 (in ethanol)^d^	
crude curcumin (38.5%)	single-stage cocrystallization by using RES as the coformer in ethanol	63.2	32.9^d^	^13^
crude curcumin (78.8%)	one-stage seeded cooling crystallization in azeotrope cosolvents of ethyl acetate/ethanol and ethyl acetate/isopropanol	94.0 ± 0.37 (in ethyl acetate/ethanol azeotrope pair)	29.0 ± 1.35 (in ethyl acetate/ethanol azeotrope pair)^e^	^14^
		94.1 ± 0.46 (in ethyl acetate/isopropanol azeotrope pair)	26.4 ± 1.30 (in ethyl acetate/isopropanol azeotrope pair)^e^	
raw turmeric powders (unknown initial CUR content)	a combination of single-stage extraction, one stage solid–liquid equilibration, and three stages of seeded cooling crystallization in water/ethanol/acetonitrile 1/44/55 (w/w/w) cosolvent	97.2	0.06^f^	^15^

a Percent crystallized per initial mass of curcumin dissolved.

b Mass of CUR in the product/initial mass of turmeric powders × 100%.

c Mass of CUR in the product/mass of postextraction materials × 100%.

d Mass of CUR in the product/mass of CUR in the starting material × 100%.

e Mass of the solid product/initial mass of crude curcumin × 100%.

f Mass of the solid product/initial mass of raw turmeric powders × 100%.

### Cocrystallization as a Means of Purification

Conventional purification techniques, such as chromatography and crystallization, possess some limitations that hinder effective purification. Chromatography often involves excessive consumption of solvents relative to the quantity of the product, limiting its application for scale-up.^16^ On the other hand, while a regular crystallization process is generally more scalable, its effectiveness can be hindered if there is any structural similarity between the product and its impurity substance, which complicates the process and reduces product yield.^12,15^

Cocrystallization has emerged as an alternative to overcoming those limitations in regular crystallization. By utilizing the intermolecular interactions between the target molecule and coformer as an additive, it provides a degree of selectivity that cannot be afforded by a simple crystallization process. The molecular interaction between the target and coformer is primarily driven by hydrogen bonding to assemble well-defined crystalline structures without altering the covalent structure of both compounds. Unlike salt formation, which relies on strong ionic interactions and requires the compound to be ionizable, cocrystallization offers a broadly applicable alternative that provides a relatively higher degree of specificity than salt.^17^

Cocrystallization as a means of purification is rooted firmly in the field of the chiral separation of active pharmaceutical ingredients. By using a chirally pure coformer, a target enantiomer from racemic mixtures can be separated from its counterpart, and unlike salt formation, it preserves the molecular structure of the target enantiomer throughout the process. For example, racemic ibuprofen and etiracetam could be resolved by cocrystallization with (S)-etiracetam^18^ and (S)-2-chloro-(S)-mandelic acid,^19^ respectively. Moreover, the utility of cocrystallization is not limited to enantiomeric purification. It has also been employed to separate a target compound from structurally similar impurities. For example, vanillin was purified by forming a 1:2 phenazine–vanillin cocrystal in toluene. Here, phenazine was found to form a cocrystal with vanillin, but not with its impurity, vanillyl alcohol. The cocrystal was later dissociated in acetone, where vanillin remained dissolved, and phenazine crystallized out after seeded cooling crystallization.^20^ The approach takes advantage of the molecular recognition of the coformer toward the target compound, thereby enabling effective purification. While the above cases represent examples where the coformer selectively binds with a compound but not the other, there are also cases where the coformer can bind with several compounds at once. For example, myricetin, quercetin, and baicalein were found to form a cocrystal with caffeine. Despite this, separation is still possible by harnessing the differences in the affinity of these cocrystals in methanol.^21^ In another example, cocrystallization was also used to isolate dihydromyricetin (DMY) from vine tea extract, which also contains the structurally similar flavonoid, myricetin (MYR).^22^ While both compounds can form cocrystals with berberine chloride (BER) as the coformer, MYR exhibits a greater propensity to cocrystallize with BER, thus enabling separation.

### This Work: Cocrystallization of Curcumin with Resorcinol

In this work, isolation and purification of curcumin (CUR) from turmeric via cocrystallization is presented. CUR has several possible suitable coformer candidates whose cocrystallizations have been verified either by binary phase diagram or single-crystal X-ray diffraction: catechol, resorcinol, hydroquinone, hydroxyquinol, and pyrogallol.^23^ This system represents an interesting case because there are at least two impurities whose chemical structure is similar to that of CUR (Figure 1).

One of the possible coformer candidates for CUR is resorcinol (RES), forming a 1:1 CUR:RES cocrystal.^24^ On the other hand, RES does not cocrystallize with BDMC,^25^ and is currently not known to cocrystallize with DMC as well to the best of our knowledge, unlike another hydroxybenzene, pyrogallol, which forms cocrystals with both CUR and BDMC.^25^ Wünsche et al.^13^ performed the cocrystallization against a crude curcuminoid mixture with an initial CUR content of 79.6% using RES in toluene/ethyl acetate 90/10 (v/v) and pure ethanol separately, resulting in cocrystals with CUR purity and yield of 83.5–86.4 and 78.4–96.4%, respectively. The authors also tested the cocrystallization against another crude curcuminoid mixture with a lower CUR content of 38.5% using pure ethanol, producing cocrystals with 63.2% purity and 32.9% yield.

Given the potential of utilizing cocrystallization for CUR purification, the following items are explored. First, a CUR–RES–solvent ternary phase diagram was constructed to determine the best pathway for purification. This is important because, depending on the solvent, the system may be congruent or incongruent, which governs the subsequent purification strategy. Once the congruency was determined, the devised strategy was tested for purifying CUR from oleoresin extracted from raw turmeric powders while also enabling the reuse of the RES coformer.

## Materials and Methods

### Chemicals

Turmeric rhizome powder, which originated in Taitung, Taiwan, was purchased from a local store. Curcumin (95% purity total curcuminoid content from turmeric rhizome, Lot: 10224826) was purchased from Alfa Aesar (US). In the subsequent section, this curcumin will be termed as “purchased CUR”, with a curcumin content of ∼70 wt % based on our preliminary analysis by HPLC. To obtain a purified CUR standard material, this purchased curcumin was further purified by seeded cooling recrystallization three times in a ternary cosolvent system of water/ethanol/acetonitrile at 1/44/55 (w/w/w) until the purity reached 98.2 wt % as determined by high-performance liquid chromatography (HPLC).^15^ This material was verified to be the crystalline Form I CUR by powder X-ray diffraction (PXRD) analysis. For the subsequent sections, this material is referred to as “CUR standard”. More details are given in the Supporting Information.

Resorcinol (RES) (99% purity, Lot: 10242296) was purchased from Thermo Scientific (UK). RES is known to exist in four polymorphic forms: α, β, γ, and δ.^26^ Among those, the α- and β-forms are stable under ambient pressure, while the γ- and δ-forms are only observed under high-pressure conditions.^26^ To avoid the issue of polymorph mixture, a temperature cycle was carried out on the RES solids before use, with the detailed procedure and characterization given in the Supporting Information as Figures S1–S3. Unless otherwise specified, any mention of resorcinol or RES for the rest of this work refers specifically to the α form.

### Solvents

Ethanol (EtOH) (99.5% purity, Lot: 103273-3) and acetonitrile (ACN) (99.9% purity, Lot: EMB4202) were purchased from Echo Chemical (Taiwan). Glacial acetic acid (99.5% purity, Lot: 31025A4001) was purchased from Uni-Onward (Taiwan). Deuterated dimethyl sulfoxide (DMSO-*d*_6_) (≥99.9% purity, Lot: A0445424) was purchased from Thermo Scientific (USA). Deuterium oxide (D_2_O) (99.8% purity, Lot: A0431395) was purchased from Acros Organics (Belgium). Methanol (MeOH) (99.9% purity, Lot: 21100053) was purchased from Tedia (USA). Reverse osmosis (RO) water was produced using a Milli-RO Plus purification system (Millipore, Billerica, MA, USA).

### Construction of T-x CUR–RES Binary Phase Diagram

DSC was employed to determine the melting points and eutectic points of various samples, which were then plotted as a T-x CUR–RES binary phase diagram. The binary phase diagram itself would then be used to quantify the composition of CUR and RES in the residual solid phase during subsequent ternary phase diagram construction.

The samples were prepared as follows: CUR standard and RES solids were mixed at different molar ratios. The CUR mole fraction ranged from 10 to 90 mol % in 10% increments, resulting in a total of nine sample groups. In each group, the combined weight of CUR and RES was fixed at 20 mg. To facilitate uniform mixing, 1 mL of anhydrous EtOH was added to each mixture. The resulting suspension was transferred into 10 mL scintillation vials and left in an oven at 60 °C overnight to allow complete evaporation of ethanol, yielding a dry and homogeneous powder mixture. Based on our trial, CUR solids remained stable at this drying temperature even for up to 3 days (^1^H NMR data are provided in the Supporting Information as Figure S4). After drying, differential scanning calorimetry (DSC) analysis was performed on all nine solid mixtures as well as on the CUR standard and RES solids. Their thermal behaviors were measured to identify the eutectic points and melting points for each composition, which were then used to construct the T-x CUR–RES binary phase diagram.

### Construction of CUR–RES–EtOH Ternary Phase Diagram

A ternary phase diagram for the CUR–RES–EtOH system was constructed by the wet residue method.^27^ The ternary phase diagram constructed in this work is drawn in a rectangular shape (Figure 2) instead of the regular triangular one for better visual clarity.^28,29^ Before the experiment, all equipment, including 7 and 20 mL vials, syringes, pipet tips, and 0.22 μm PVDF filter membranes, was oven-dried at 60 °C for 1 h to eliminate moisture interference and subsequently weighed. Solid powders of CUR standard and RES with known mole fractions were suspended in EtOH, where the mole fraction is defined in eq 1. EtOH was also accurately weighed and converted to moles to correspond to a predetermined *Q* value, defined as the mole ratio of solvent-to-other components, given in eq 2. The mole fraction of the CUR and *Q* values defined the initial point coordinates in the phase diagram construction (Point I in Figure 2). All suspensions were equilibrated at 25 °C in a water bath for 72 h. The mother liquor was carefully separated from the suspension by a micropipette and then filtered through a 0.22 μm PVDF filter in the 20 mL vial. It was subsequently oven-dried at 60 °C until a constant weight was achieved. The dried solid residue obtained from the mother liquor was analyzed using HPLC to quantify the CUR content, while the RES quantity was determined by subtracting the CUR content from the initial mass of the sample. Based on the mole number of evaporated EtOH, CUR (from HPLC), and RES (by mass balance), the *Q* value and mole fraction from the mother liquor of the tie-line could be calculated as Point M in Figure 2.

$$\mathrm{Mole}\;\mathrm{fraction}=\frac{\mathrm{Mole}\;\mathrm{of}\;\mathrm{CUR}}{\mathrm{Mole}\;\mathrm{of}\;\mathrm{CUR}+\mathrm{Mole}\;\mathrm{of}\;\mathrm{RES}}$$ 1

$$Q=\frac{\mathrm{Mole}\;\mathrm{of}\;\mathrm{EtOH}}{\mathrm{Mole}\;\mathrm{of}\;\mathrm{CUR}+\mathrm{Mole}\;\mathrm{of}\;\mathrm{RES}}$$ 2

**Figure 2:**
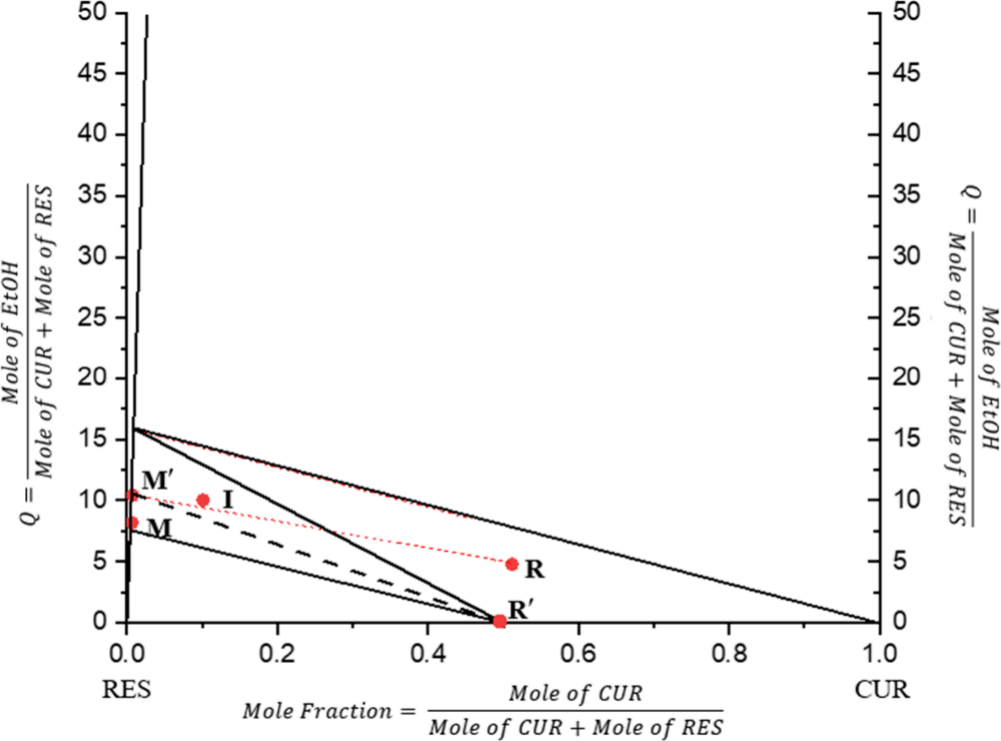
Procedure for constructing CUR–RES–EtOH ternary phase diagram at 25 °C and 1 atm. Points I, M, and R represent the experimental composition of the starting condition, mother liquor phase at equilibrium, and wet solid residue at equilibrium, all determined by HPLC and mass balance. Points R′ are the equilibrium composition of the solid residue as determined by DSC by assuming a negligible cake wetting effect. Point M′ denotes the normalized mother liquor composition. The dotted red line indicates the linear regression of Points I–R, which was used to determine Point M′. The dashed black line represents the equilibrium tie-line by connecting Points M′ and R′.

The moist solid residue was similarly oven-dried at 60 °C until constant weights were achieved. The dried solid residue was analyzed using the same procedure as that for the mother liquor: CUR content was quantified by HPLC, while RES content was calculated via a mass balance. Based on those values, the *Q* value and mole fraction were calculated in the same manner. After that, Point R in Figure 2 was obtained.

### Tie-Line Regression

While Points I, M, and R obtained by the above procedure form a single equilibrium tie-line, the significantly high solubility of RES in EtOH would lead to compositional deviation due to the overestimation of the cake wetting. As illustrated in Figure 2, regressing these three points by a straight line would cause the dry solid composition to be extrapolated outside the range of mole fraction (>1.0). This deviation happened in every tie-line measured for this system. Therefore, the CUR:RES composition of the dried solid residue was analyzed by DSC instead. Here, the T-x binary phase diagram provides a reference for the relationship between the endothermic peaks and CUR:RES composition. The composition was then plotted as Point R′ in Figure 2. Note that the *Q* value of Point R′ is zero, since it was assumed to represent the composition of the completely dry solid residue.

Separately for the mother liquor composition, since there was a potential for Point R overestimation, the composition was normalized by taking this into account instead of directly applying Point M at its nominal value. To do this, Points M, I, and R were regressed in a linear plot. Afterward, the mole fraction of the mother liquor (Point M) was inserted into the regression equation to obtain the normalized *Q* value for the mother liquor phase. This normalized composition for the mother liquor phase is represented as Point M′ in Figure 2. The equilibrium tie-line was then determined by connecting Points M′ and R′. By repetition of this process across various initial compositions and *Q* values, a CUR–RES–EtOH ternary phase diagram was established.

### Workflow for CUR Extraction and Purification

The schematic workflow for curcumin purification is illustrated in Figure 3. The process began with the extraction of oleoresin from turmeric powder using EtOH with a solid-to-solvent ratio of 0.5 g/mL, which was stirred at 300 rpm for 1 h. This and all subsequent stages were conducted at 25 °C, 1 atm, and a constant stirring rate of 300 rpm. Solid–liquid equilibration was then performed by using an oleoresin-to-EtOH ratio of 0.316 g/mL for 8 h to yield crude curcumin powders.

**Figure 3:**
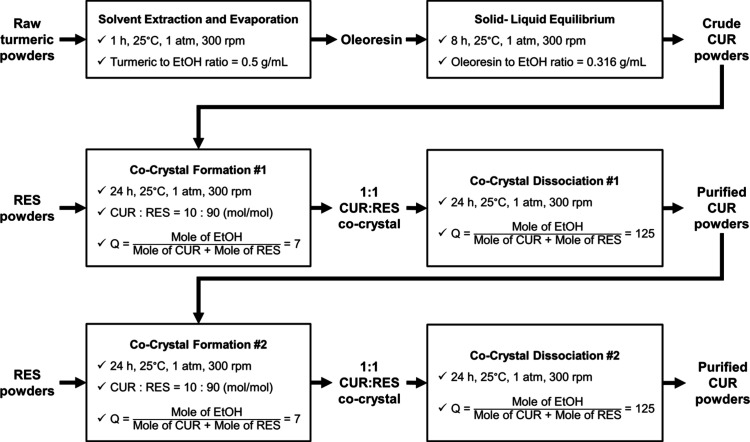
Schematic workflow from turmeric extraction to cocrystal dissociation. The purification process for curcumin consists of several major stages, all performed at 25 °C, 1 atm, and stirred at 300 rpm: (1) extraction, where turmeric powder was extracted using EtOH with a solid-to-solvent ratio of 0.5 g/mL for 1 h, yielding oleoresin, (2) solid–liquid equilibrium, where oleoresin was suspended in EtOH at a ratio of 0.316 g/mL and stirred for 8 h, enhancing curcumin purity to obtain crude curcumin powders, (3) cocrystal formation, where crude curcumin was subjected to cocrystallization with resorcinol in a CUR:RES mole ratio of 1:9 under a *Q* value of 7, and (4) cocrystal dissociation, where the cocrystal was resuspended in EtOH with *Q* increased to 125 and stirred for 24 h, resulting in purified curcumin. Steps (3) and (4) were repeated to obtain CUR purity of at least 95 wt %.

The reason RES was not used for extraction and the equilibration step was that at these two steps the quantity of impurities predominates, while the constructed ternary phase diagram in Figure 6 was based on pure CUR powders. Attempting to apply this phase diagram in such conditions could produce unexpected outcomes. For example, using RES on oleoresin (CUR purity ≤ 30 wt %) caused a complete dissolution of the solids, despite the original aim to form the 1:1 cocrystal. Therefore, the phase diagram can only be used when the quantity of CUR is more than its impurities. From our experience, the phase diagram is applicable if the CUR purity is at least 70 wt % or above.

Once the crude CUR powders were obtained from the oleoresin, it was subjected to cocrystallization with RES. Based on the CUR–RES–EtOH ternary phase diagram (which will be discussed in the Results and Discussion section), crude curcumin was then cocrystallized with the RES:CUR mole ratio set at 9:1 and a *Q* value of 7. Based on preliminary investigation in Figure S5, stirring was maintained for at least 24 h to attain an equilibrium state, producing a single solid phase of 1:1 CUR–RES cocrystals. The filter cake after the cocrystal formation step was not washed to prevent premature dissociation. In the subsequent dissociation step, the 1:1 CUR:RES cocrystals were suspended in EtOH at the *Q* value of 125 under continuous stirring for 24 h, thereby shifting the system toward the curcumin-rich region in the CUR–RES–EtOH ternary phase diagram. Upon equilibrium, purified CUR powders were obtained. The postdissociation filter cake was washed via dropwise addition of about 1 mL of EtOH for each milligram of the cake during filtration to remove residual RES. Afterward, the process of cocrystal formation and dissociation was repeated again to reach a CUR purity of ≥95 wt %.

### Workflow for RES Recovery and Reuse

The process flow diagram for RES recovery and reuse is illustrated in Figure 4. The procedure for the cocrystal formation and dissociation is exactly the same as the one in Figure 3. However, since cocrystallization is only effective when the quantity of CUR is more than its impurities, this study was carried out against the purchased CUR powders with a starting purity of ∼70 wt % instead of moving back to turmeric powders.

**Figure 4:**
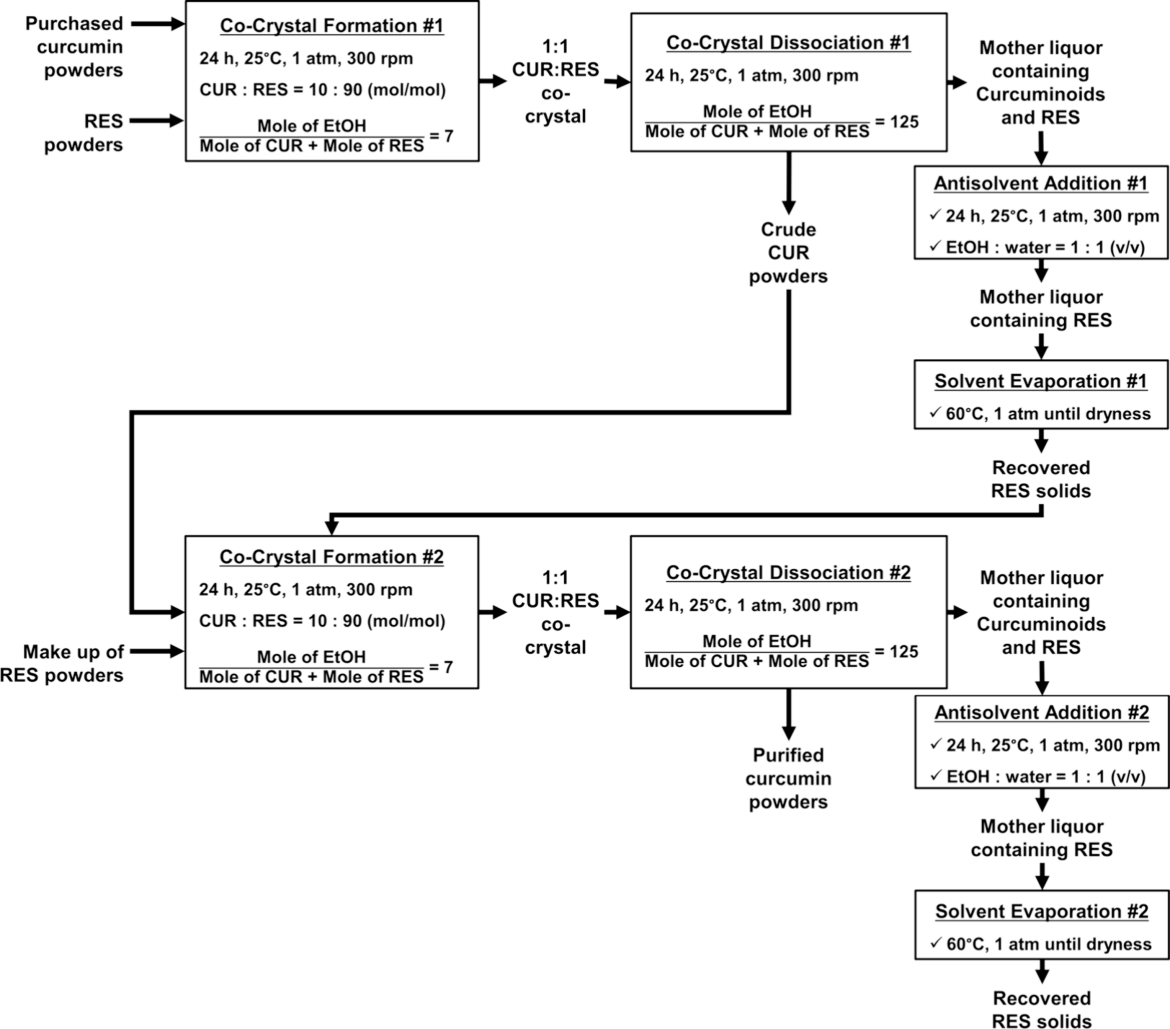
Schematic workflow of the purification of CUR from the purchased CUR and the subsequent recovery and reuse of RES. The cocrystallization procedure was identical with the one in Figure 3. Recovery of RES was carried out by antisolvent precipitation of the curcuminoids in the mother liquor from the cocrystal formation step, followed by evaporation until dryness.

Given that the mother liquor obtained postcocrystal formation should be enriched with RES, the recycling step was carried out on it. However, this mother liquor also contains some curcuminoids, which must be removed to prevent adulteration of the recovered RES solids. The removal of these impurities from the mother liquor was facilitated by the antisolvent addition using water. Based on the solubility curve of the purchased CUR powders at different EtOH:water volume ratios (Figure S6), the ratio of 1:1 (v/v) was deemed to be sufficient since it should be able to precipitate almost all of the solutes but not RES. Afterward, the filtered mother liquor was subjected to evaporation until dryness to recover the RES solids for subsequent reuse.

### Direct CUR Purification by RES as an Additive

Instead of treating RES as a coformer that crystallizes together with CUR as a solid cocrystal, this experiment was conducted by solubilizing all of RES into a CUR–EtOH suspension. First, RES was dissolved in EtOH. Afterward, the ethanolic solution of RES was introduced and equilibrated with purchased CUR powders (∼70 wt % purity) to obtain a suspension with the overall composition of 1:1 CUR:RES (mol/mol) and the solid-to-solvent ratio of 0.273 g/mL. The suspension was stirred at 300 rpm and 25 °C for 72 h to ensure equilibrium could be attained. Based on the ternary phase diagram, which will be discussed in the Results and Discussion section, RES is completely dissolved, while some CUR solids remain. The solids were filtered, followed by filter cake washing by the dropwise addition of a small quantity of EtOH. The cake was then oven-dried. For a control experiment, another experiment was also conducted under identical procedures but without RES. All experiments were performed in triplicate.

### Differential Scanning Calorimetry (DSC)

DSC was employed to measure the melting temperature and enthalpy values. Approximately 2 mg of the sample was placed in a standard aluminum pan with a perforated lid. Heating was done under a continuous nitrogen flow with a flow rate of 20 mL/min and 99.990% purity. Thermal analysis was performed using a Perkin-Elmer DSC-7 calorimeter (Perkin-Elmer Instruments LLC, Shelton, CT, USA) with a constant heating rate of 2 °C/min for the construction of the T-x binary phase diagram or 10 °C/min for other samples.

### High-Performance Liquid Chromatography (HPLC)

Quantitative analysis of the purity and concentration of CUR in the samples was performed using a Shimadzu Prominence-i LC-2030C 3D Plus instrument (Shimadzu, Japan), while the quantity of RES was determined by mass balance. Samples were dissolved or diluted in methanol to achieve a concentration range of 0.05–0.2 mg/mL. All samples were filtered using a 25 mm PVDF 0.22 μm hydrophilic syringe filter before injection. Chromatographic separation was carried out using a Phenomenex Kinetex C18 column (particle size: 5 μm; pore size: 100 Å; dimensions: 150 × 4.6 mm). The mobile phase consisted of 2% (v/v) aqueous acetic acid and acetonitrile at a volume ratio of 60:40 (v/v), where the 2% (v/v) acetic acid solution was prepared by a 50-fold dilution of glacial acetic acid. An isocratic flow rate of 0.8 mL/min was maintained, and the column temperature was kept at 30 °C. Each injection volume was 20 μL; detection was carried out at a UV wavelength of 425 nm. This HPLC analytical procedure and the internal calibration line used were identical to our previous work.^15^

### Nuclear Magnetic Resonance (NMR)

The qualitative purity and molecular structure of the purified CUR powders and recovered RES samples were confirmed by NMR spectroscopy using a Bruker Ascend 600 MHz NMR spectrometer (Bruker, Germany). ^1^H and ^13^C NMR spectra were recorded, with chemical shifts providing functional group identification and signal intensities allowing estimation of atomic ratios. NMR sample solutions of CUR and RES were prepared separately at a concentration of 39 mg of CUR in 0.5 mL of DMSO-*d*_6_ and 220 mg of RES in 1.5 mL of D_2_O, respectively.

### Optical Microscopy (OM)

For microscopic observation of crystal habit, CUR and RES solids were dispersed in silicone oil, while 1:1 CUR:RES cocrystal samples were directly collected from the mother liquor upon cocrystallization. A small volume of the suspension was transferred onto a slide and covered with a coverslip. Optical micrographs were obtained using an Olympus BX-51 microscope (Olympus Corp., Tokyo, Japan) equipped with a Moticam 2000 digital camera (Motic, Hong Kong, China) and a cross-polarized filter. Images were captured and processed using Motic Images Plus software (version 2.0).

## Results and Discussion

### Binary Phase Diagram of CUR:RES

The binary and ternary phase diagrams of CUR–RES and CUR–RES–EtOH are shown in Figures 5 and 6, respectively. The existence of the 1:1 CUR:RES cocrystal was verified by the presence of a new melting temperature at a 1:1 mol ratio and two eutectic melting compositions at the CUR mole fraction of 0.1 and 0.6.

**Figure 5:**
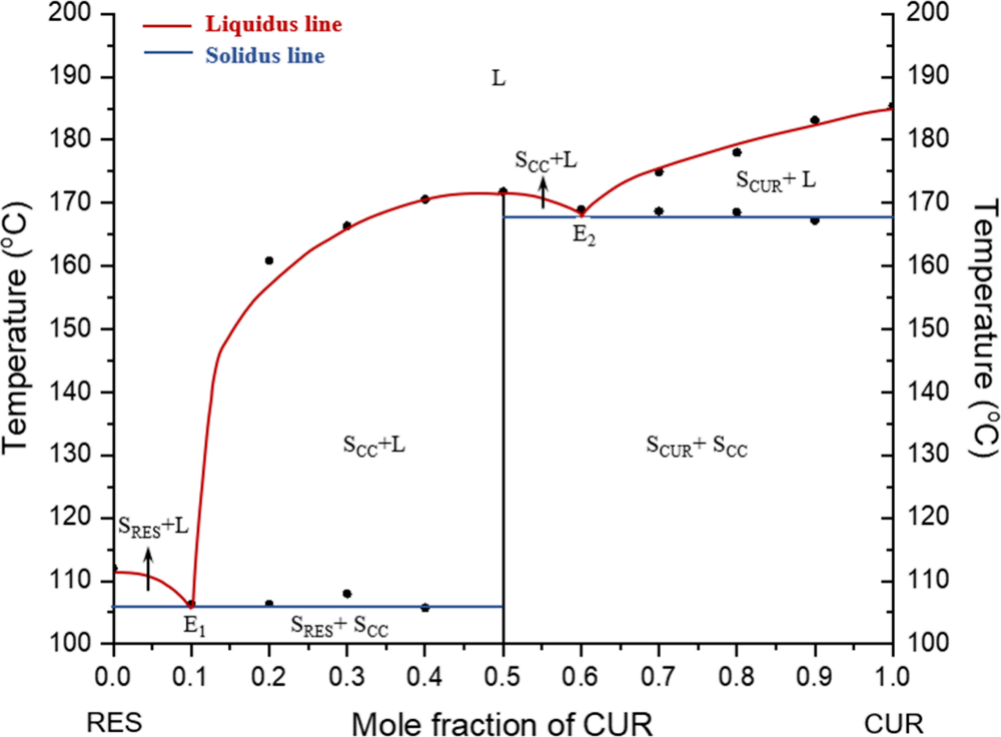
Binary phase diagram of the CUR–RES system constructed by using peak melting temperatures obtained from DSC analysis. The *x*- and *y*-axis represent the mole fraction of CUR and temperature, respectively. The red curves represent the liquidus lines, showing the melting for mixtures with increasing CUR content; the horizontal blue lines represent the solidus lines. L: liquid, S_RES_: solid of resorcinol, S_CUR_: solid of curcumin, S_CC_: solid of cocrystal, E_1_: eutectic point of resorcinol with 1:1 CUR:RES cocrystal, and E_2_: eutectic point of curcumin with 1:1 CUR:RES cocrystal. DSC scans for each data point are provided in Figure S7 in the Supporting Information.

**Figure 6:**
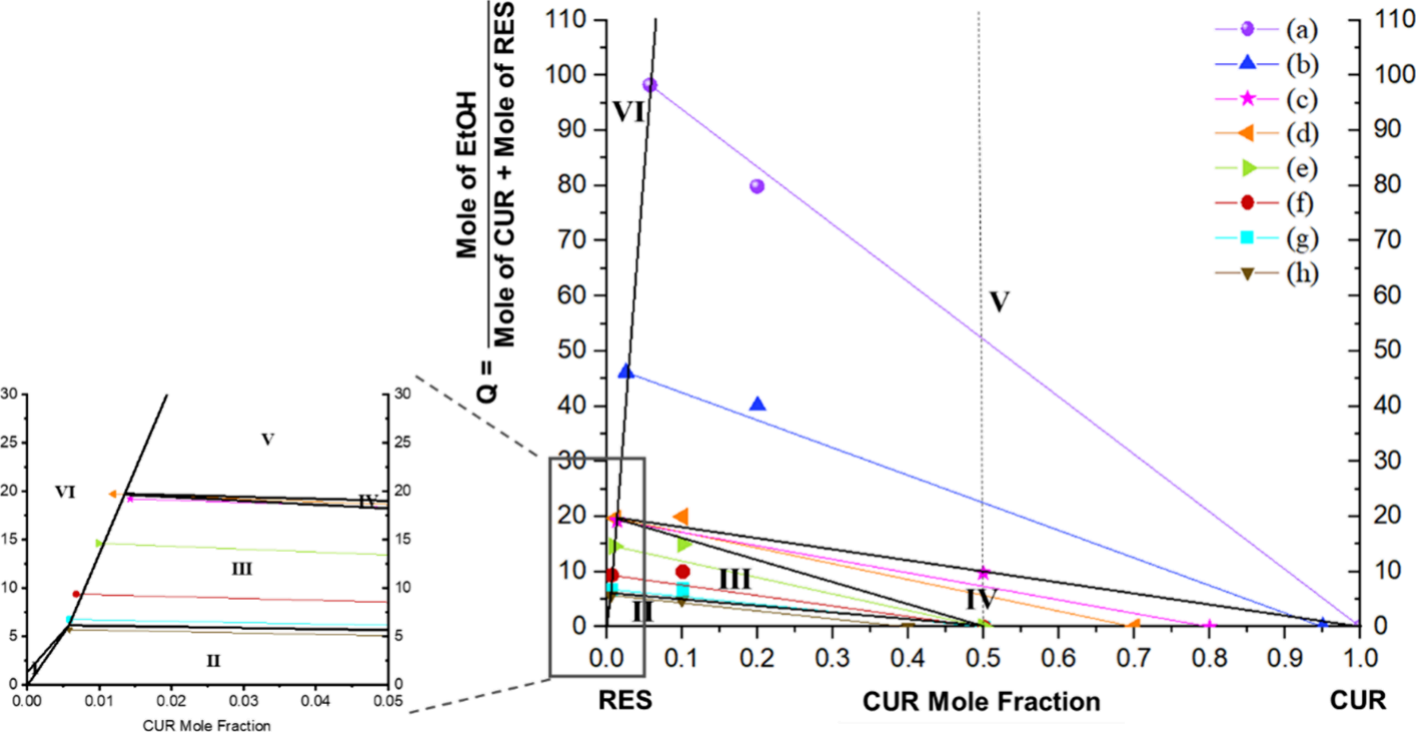
Ternary phase diagram of the CUR–RES–EtOH. The phase diagram comprises six regions based on the solid–liquid phase equilibria, Region I: RES (s) + liquid solution; Region II: RES (s) + cocrystal (s) + liquid solution, Region III: cocrystal (s) + liquid solution, Region IV: CUR (s) + cocrystal (s) + liquid solution, Region V: CUR (s) + liquid solution, and Region VI: Liquid solution only. Solid black lines are the phase boundaries. Colored thin solid lines of (a–h) are equilibrium tie-lines, with the detailed composition for each point provided in the Supporting Information. The dashed vertical line is a 1:1 stoichiometric line. Equilibrium data points are provided in Table S1 in the Supporting Information.

### Ternary Phase Diagram of CUR:RES:EtOH

In contrast to the binary phase diagram, the ternary phase diagram is highly asymmetrical, where the mole ratio of the two ternary invariant points is completely distorted from the eutectic composition according to the binary phase diagram, skewing far toward the RES side at CUR mole fraction (with respect to CUR and RES) of 0.006 and 0.014. The 1:1 molar stoichiometric line does not intersect the 1:1 CUR:RES cocrystal (s) + liquid solution region (Region III in Figure 6) of the diagram, a trait of an incongruent cocrystal ternary phase diagram,^30^ which is highly dependent on the solvent used.^31^ This highly asymmetric incongruency means that the cocrystal cannot be obtained from an equimolar ratio of CUR:RES in ethanolic solution. Instead, an excess amount of RES is necessary to bring the system to the cocrystallization region (Region III in Figure 6) in a suitable quantity of EtOH. For the subsequent cocrystal formation, a CUR:RES mole ratio of 1:9 and a *Q* value of 7, which are located within Region III, were adopted.

Instead of being a burden, this incongruent behavior provides a practical advantage for the cocrystal dissociation. In order to dissociate the cocrystal, the system has to enter Region V in Figure 6. Because Region V spans a wide compositional range, one only needs to introduce EtOH to the 1:1 CUR:RES cocrystal solids until the composition is inside this region. Once the cocrystal enters this region, the dissociation would happen instantaneously, producing purified CUR powders.

### Extraction and Solid–Liquid Equilibration

In order to provide a full picture of the isolation of plant-based compounds, this work used raw turmeric powder as the starting material. Therefore, extraction procedures were carried out before purification. Ethanol was used since it belongs to Class 3 solvent according to the ICH Guideline for Residual Solvents, which is deemed to be the least toxic solvent class. RES was not necessary for the extraction step because this step aims to separate CUR from the rest of the turmeric root solid powders, making its usage redundant. After the extractant solution was recovered and the solvent evaporated, tacky solids of oleoresin, which comprised curcuminoids, essential oils, fats, fatty acids, and other impurities, were obtained. The average CUR content and oleoresin yield from three repeated experiments were 21.26 ± 0.83 and 5.68 ± 0.40 wt %, respectively, as summarized in Table 2.

**Table 2:** Results of the CUR Isolation from Powdered Turmeric According to the Procedure in Figure 3 ^a^,^b^,^c^

**stage**	**CUR purity (wt %)**	**yield (wt %)**
turmeric extraction	21.26 ± 0.83	5.68 ± 0.40
solid–liquid equilibration	74.74 ± 1.26	16.14 ± 1.48
cocrystal formation and dissociation #1	89.4 ± 2.94	50.99 ± 3.13
cocrystal formation and dissociation #2	96.06 ± 1.19	70.23 ± 3.26

a The average and standard deviation of CUR purity and yield values at each stage were based on data obtained from three independent repetitions. The exact values for each repetition are attached in Tables S2–S4 in the Supporting Information.

b CUR purity is calculated by 
$$\mathrm{CUR}\;\mathrm{purity}\;(\mathrm{wt}\;\%)=\frac{\mathrm{Mass}\;\mathrm{of}\;\mathrm{CUR}}{\mathrm{Total}\;\mathrm{mass}\;\mathrm{of}\;\mathrm{sample}}\times 100\%$$

c Yield is calculated by 
$$\mathrm{Yield}\;(\mathrm{wt}\;\%)=\frac{\mathrm{Output}\;\mathrm{solids}\;(g)}{\mathrm{Input}\;\mathrm{solids}\;(g)}\times 100\%$$

For the next step, oleoresin was purified by solid–liquid equilibration in ethanol. At this stage, the purification by cocrystallization with RES was not feasible because the curcumin content was only about 21 wt %, much less than the impurities. Our preliminary trial by introducing RES and EtOH to induce the system to enter Region III in Figure 6 only resulted in complete dissolution. Since the ternary phase diagram was built based on the CUR standard, a situation where CUR is the minority species, such as in the oleoresin, made the ternary phase diagram unusable. Hence, only ethanol was used for the first purification step, similar to the procedure in our previous work.^15^ As expected, this procedure could purify CUR further to 74.74 ± 1.26 wt % with the solids yield of 16.14 ± 1.48 wt % after three experiments, as tabulated in Table 2.

### Cocrystal Formation and Dissociation

After the preliminary solvent extraction and solid–liquid equilibrium, crude curcumin solids were obtained. This crude product was then introduced into a slurry crystallization process conducted in Region III of the CUR–RES–EtOH ternary phase diagram in Figure 6. Using the phase diagram as a guide, Region III was identified as the domain where the 1:1 CUR:RES cocrystal is the only solid phase in equilibrium with the solution. To ensure a successful cocrystallization, the operational point (*Q* = 7 and CUR mole fraction = 10 mol %) was deliberately chosen within the broadest area of the triangular Region III to minimize the risk of unintentionally shifting into adjacent regions, especially by taking into account the presence of impurities. Although the CUR–RES system exhibits strong incongruent solubility behavior, the cocrystal can still form as long as the overall composition lies within Region III. A change of color was observed from yellow to orange, suggesting that cocrystallization was indeed taking place.

Once the cocrystals were harvested, they were subsequently guided into Region V of the phase diagram in Figure 6 to initiate the cocrystal dissociation. Owing to the incongruent behavior of the system, dissociation can be initiated just by simply suspending the 1:1 CUR–RES cocrystal solids in a suitable amount of EtOH, something that is not possible if the system is congruent. At the end, a high *Q* value (*Q* = 125) was chosen to drive the system far above the phase boundary line as a margin of safety to ensure that no RES or other impurities could be carried over in the solid phase. The CUR purity and the yield of the solids from three repeated experiments are 89.40 ± 2.94 and 50.99 ± 3.13 wt %, respectively. The purified CUR solids were then subjected to the identical treatments of cocrystallization and dissociation, further improving the CUR purity to 96.06 ± 1.19 wt %. The overall extraction and purification yield from the turmeric powders, all the way down to the purified CUR solids, averaged about 0.26 ± 0.02 wt %. Qualitative analysis by ^1^H and ^13^C NMR (Figures 7 and 8) indicates that only CUR is present without any detectable residual RES.

**Figure 7:**
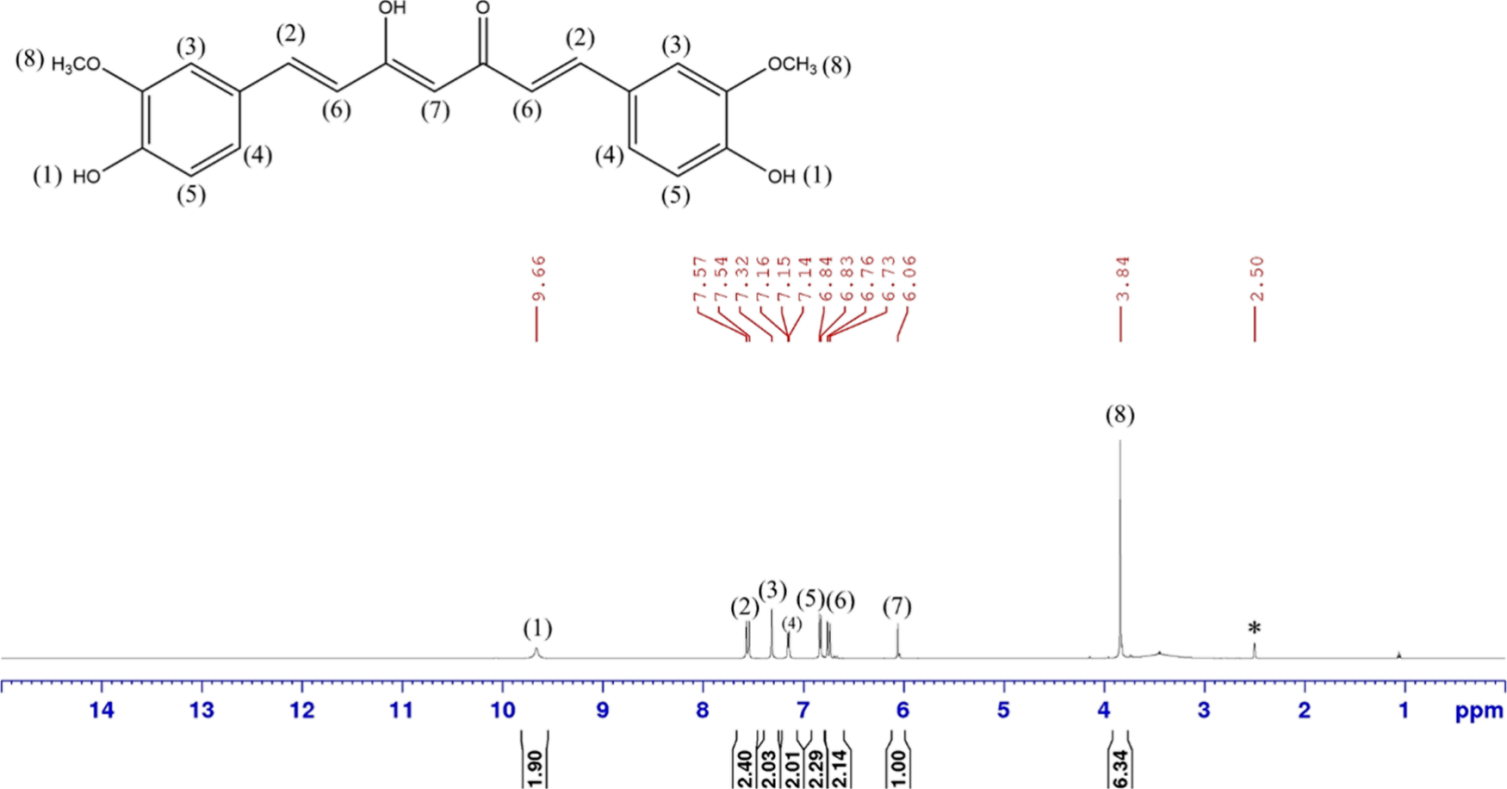
^1^H NMR spectrum of purified CUR in DMSO-*d*_6_. The asterisk (*) indicates the residual solvent peak of DMSO-*d*_6_.

**Figure 8:**
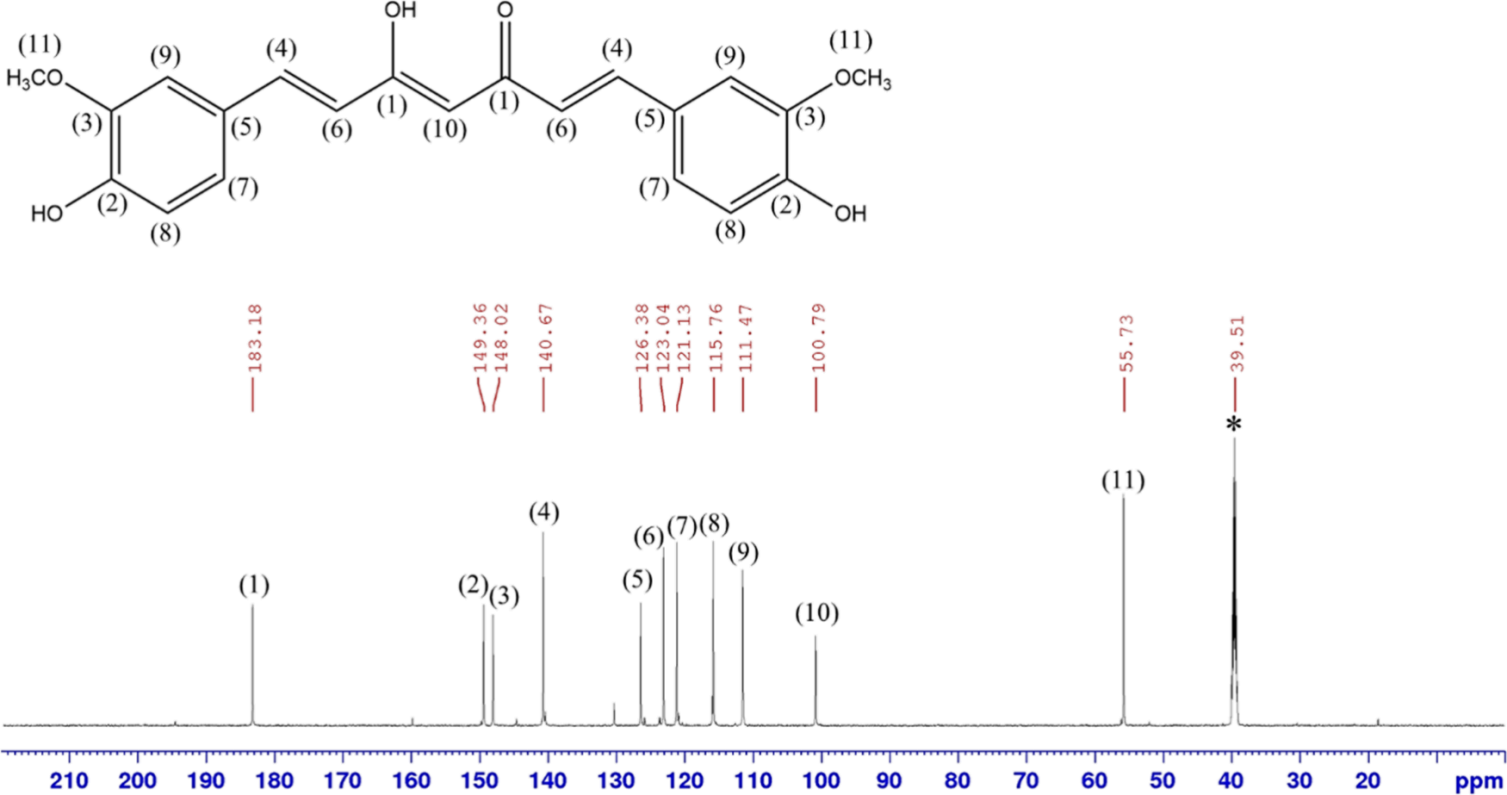
^13^C NMR spectrum of purified CUR in DMSO-*d*_6_. The asterisk (*) indicates the residual solvent peak of DMSO-*d*_6_.

### Coformer Recycling

Three repeated experiments of curcumin purification were conducted in this study to evaluate the feasibility of performing RES recovery and reuse. An identical procedure as above was repeated again, except that this time, purchased CUR powders (purity ∼ 70 wt %) were used instead of starting back from turmeric powders.

In the first cocrystal formation and dissociation stage, CUR purity and yield of 91.3 ± 1.77 and 64.2 ± 1.30 wt % were obtained, respectively. On average, the volume of the mother liquor separated after the cocrystallization was 31.0 ± 1.73 mL for the three repeated experiment batches. An equal volume of water was added as an antisolvent to induce precipitation of the curcuminoid components that did not participate in the cocrystal formation. After precipitation, filtration, and evaporation of the mother liquor, 7589.6 ± 129.7 mg of RES was recovered, corresponding to a recovery yield of 80.6 ± 1.40 wt %. These data are presented in Tables 3 and S5 in the Supporting Information. ^1^H and ^13^C NMR spectra of the recycled RES (attached in the Supporting Information as Figures S13 and S14, respectively) do not show additional peaks besides RES.

**Table 3:** Results of the CUR Purification from Purchased CUR Powders by Enabling RES Recovery and Reuse Procedure Are Shown in Figure 4 ^a^,^b^

**stage**	**CUR purity (wt %)**	**CUR yield (wt %)**	**qualitative RES purity**	**RES yield (wt %)**
cocrystal formation, dissociation, and RES recovery #1	91.3 ± 1.77	64.2 ± 1.30	verified by ^1^H and ^13^C NMR	80.6 ± 1.40
cocrystal formation, dissociation, and RES recovery #2	96.0 ± 1.11	71.2 ± 0.42	verified by ^1^H and ^13^C NMR	78.1 ± 2.44

a The yield was calculated as the ratio of the weight of the final product to that of the raw material.

b The average and standard deviation of CUR purity and yield values at each stage were based on data obtained from three independent repetitions. The exact values for each repetition are attached in Tables S5 and S6 in the Supporting Information.

To further enhance the purity of CUR, the RES solids obtained from the first stage of cocrystal purification were used as the starting material for a second-stage slurry cocrystallization process. In this stage, the recycled RES obtained from the previous procedure was reintroduced as the coformer, enabling material recycling. However, using recycled RES alone was insufficient to meet the required 1:1 stoichiometric ratio for CUR–RES cocrystal formation. Therefore, additional RES was supplemented to achieve the ideal composition and ensure successful cocrystal formation.

The CUR sample obtained from the second stage process was analyzed by HPLC and found to have a purity of 96.0 ± 1.11 wt %, representing an improvement over the 91.3 ± 1.77 wt % achieved in the first stage. In addition, the yield slightly increased from 64.2 ± 1.30 wt % in the first stage to 71.2 ± 0.42 wt % in the second stage, with the relevant data summarized in Tables 3 and S6 in the Supporting Information. This increase in yield is likely attributable to the higher purity of the CUR starting material used in the second stage, which may have improved the cocrystal formation efficiency and, consequently, the final recovery percentage. These results demonstrate that a multistage cocrystal purification strategy provides an effective stepwise enhancement of purity. Furthermore, the reuse of recycled RES contributes to improved resource efficiency and a reduction in the overall process cost.

### Crystal Morphology

Figure 9 displays the crystal morphology evolution at different time points during the slurry cocrystallization process, with images captured using OM at different time points up to 48 h. The observations revealed that after 2 h, a small number of red prismatic crystals began to appear, indicating the initial formation stage of the 1:1 CUR–RES cocrystal. As time progressed, the number of crystals gradually increased, and by 24 h, a large quantity of cocrystals appeared. The crystal size continued to grow, and by 48 h, well-developed, macroscopically distinguishable crystals were clearly formed. Furthermore, the overall crystal appearance remained consistent throughout the process, exhibiting uniform, well-defined prismatic structures. No evidence of agglomeration or heterogeneous morphologies was observed. These results suggest that the cocrystal formation in this system is primarily governed by homogeneous nucleation,^32^ where new cocrystal nuclei form on the diffuse layer close to the solution phase instead of being initiated right at the surface of the existing crystals.

**Figure 9:**
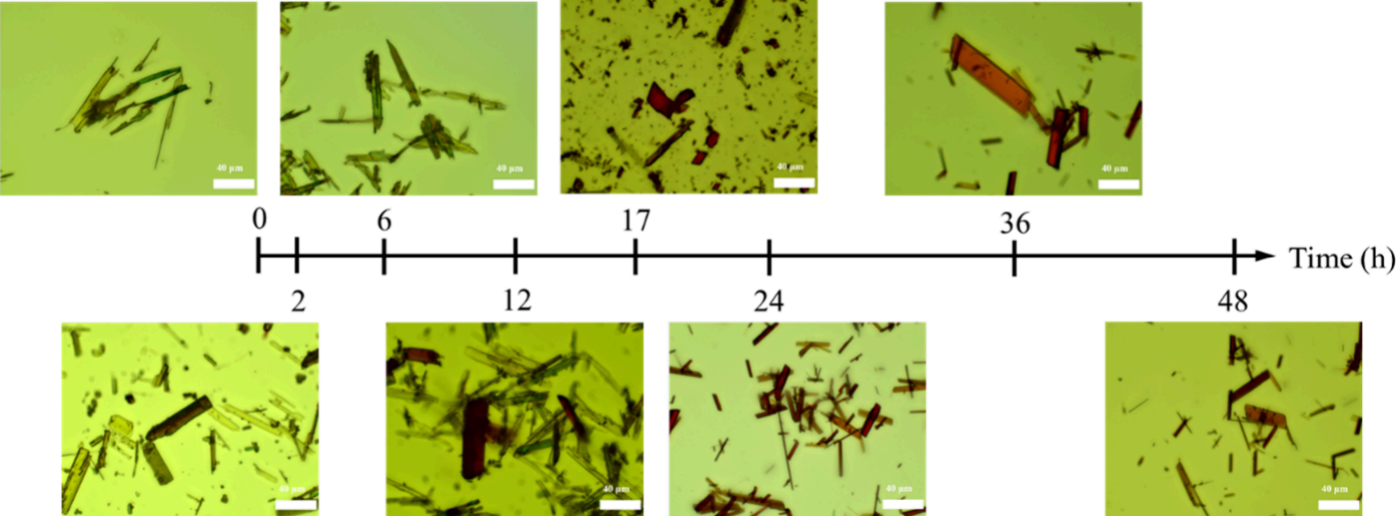
OM images showing the timeline of slurry cocrystallization. Scale bar: 50 μm.

### Direct CUR Purification by RES as a “Coformer Additive”

A single cocrystallization stage of the above-mentioned CUR purification process is essentially a two-step process, which involves separate cocrystal formation in Region III and dissociation in Region V in Figure 6. This raises the following question: to purify CUR, is it possible to just proceed directly to Region V without needing to form a cocrystal in the first place? Essentially, RES is treated simply as an additive material instead of a cocrystal former. To investigate this possibility, an experiment was carried out by suspending purchased CUR powders with an initial purity of ∼70 wt % into an ethanolic solution containing RES. The final composition after mixing was located at the operational point of *Q* value of 20 and CUR mole fraction of 0.5, well within Region V in the ternary phase diagram in Figure 6. A control experiment was also carried out with an identical procedure but without an RES.

As shown in Table 4, CUR obtained by the “RES additive” method possessed a purity of 85.3 ± 2.0 wt %, significantly higher than the simple equilibration without RES of 78.5 ± 1.6 wt %, while retaining similar crystal yields of 89.1 ± 0.7 against 89.6 ± 0.6 wt %, respectively. This result is remarkable, as simply using RES as an additive could provide a much higher purity without additional CUR loss. While the “RES additive” method gave a lower purity than the proper cocrystal formation and dissociation, it should be noted that the solids in the latter method were essentially subjected to fresh EtOH twice due to the separate cocrystal formation and dissociation steps. This is reflected in its lower crystal yield of 64.2 ± 1.30 wt %.

**Table 4:** Comparison of the CUR Purification Methods Presented in This Study with Several of Our Group’s Past Studies^a^

**initial CUR purity** (wt %)	**method**	**final CUR purity (wt %)**	**yield (wt %)**	**reference**
78.8	**unseeded cooling recrystallization**	93.5 ± 0.47	41.3 ± 3.93	experiment 2 in ref 14
	• cooling recrystallization from 65 to 25 °C at 10 °C/h, then left to stand at 25 °C for 3 days			
	• no seed addition			
	• solvent-to-solid loading: 59.3 g EtOH/g crude CUR			
	• stirring at 300 rpm			
74.2	**seeded cooling recrystallization**	93.3	37.6	stage #4 of 1–2–3 mode experiment in ref 15
	• cooling recrystallization from 60 to 25 °C at 10 °C/h, then left to stand at 25 °C for 24 h			
	• 1 wt % of high-purity CUR seeds were added at 50 °C during cooling			
	• solvent-to-solid loading: 69.2 g EtOH/g crude CUR (excluding seed)			
	• stirring at 300 rpm			
70.0	**simple solid–liquid equilibration**	78.5 ± 1.6	89.6 ± 0.6	Direct CUR Purification by RES as a “Coformer Additive” section of this study
	• solid–liquid equilibration at 25 °C for 8 h			
	• solvent-to-solid loading: 3.50 g EtOH/g crude CUR			
70.0	**RES as “coformer additive”**	85.3 ± 2.0	89.1 ± 0.7	Direct CUR Purification by RES as a “Coformer Additive” section of this study
	• solid–liquid equilibration at 25 °C for 8 h			
	• CUR-to-RES ratio: 1 g crude CUR to 0.21 g RES			
	• solvent-to-solid loading: 2.89 g EtOH/(g crude CUR + RES)			
70.0	**cocrystallization method**	91.3 ± 1.77	64.2 ± 1.30	Purification stage #1 in Tables 3 and S5 of this study
	(1) cocrystal formation			
	• equilibrating CUR–RES at 25 °C for 24 h			
	• CUR-to-RES ratio: 1 g crude CUR to 1.88 g RES			
	• solvent-to-solid loading: 2.125 g EtOH/(g crude CUR + RES)			
	• stirring at 300 rpm			
	(2) cocrystal dissociation			
	• suspending the cocrystal in EtOH at 25 °C for 24 h			
	• solvent-to-solid loading: 24.1 g EtOH/g cocrystal			
	• stirring at 300 rpm			

a All of the listed methods were carried out in EtOH. Yield is defined as the ratio of the weight of the final product to that of the raw material, i.e., initial crude CUR.

Comparison of conventional crystallization methods was also made in Table 4 against our group’s past experiments to ensure that the influences of less obvious external factors, which might subtly influence purity and yield, such as geographic ambient conditions and nonprimary procedures (e.g., filtration, cake washing, and drying), can be minimized. All of the listed items were carried out in EtOH to ensure fair benchmarking. It can be seen that compared with seeded and unseeded crystallization procedures, the cocrystallization method provides similar purity values despite the lower purity of the initial crude CUR. Moreover, the crystal yield was also up by more than 50% despite being subjected to two steps of separate cocrystal formation and dissociation. Another interesting part is in the “RES additive” method. Despite its lower purity than the seeded/unseeded crystallizations, its yield value exceeded a 100% increase, while using twenty times less amount of EtOH per gram of initial solids. It was remarkable that simply using RES as an additive could provide a huge improvement, even on par with the regular cocrystallization method. It highlights that treating the coformer as an additive is a promising alternative to cocrystallization, warranting more investigation in the future. Nonetheless, phase diagram construction remains essential, regardless of which method is used.

## Conclusions

In this work, a phase-diagram-guided cocrystallization strategy to purify curcumin was developed, employing EtOH as the solvent and resorcinol as the coformer. Starting from turmeric powders, a series of steps involving solvent extraction, solid–liquid equilibrium, and two successive cycles of cocrystal formation and dissociation were integrated, achieving a significant increase in purity in six steps. Precise control of the system composition for cocrystal formation and dissociation was enabled by the construction of the CUR–RES–EtOH ternary phase diagram at 25 °C and ambient pressure. Each operation was deliberately positioned within a specific region of the phase diagram, allowing us to traverse the desired phase boundaries and accomplish an efficient separation. This strategy effectively enabled the purification of curcumin with high selectivity and efficiency, achieving 96.06 ± 1.19 wt % CUR purity, with the overall yield from the turmeric powders all the way to the purified CUR solids being 0.26 ± 0.02 wt %. To ensure raw material recyclability in subsequent processes, an RES recycling procedure was developed. Experimental results showed that RES could be recovered at 80.6 ± 1.40 and 78.1 ± 2.44 wt %. Moreover, it was found that RES coformer could simply be introduced to a suspension of CUR in EtOH as an additive, skipping the cocrystallization and dissociation steps, producing solids with CUR purity of 85.3 ± 2.0 wt %, which is higher than simply equilibrating the solids in EtOH without RES with similar crystal yield.

It is worth mentioning that while both the cocrystallization and “coformer additive” methods were feasible and demonstrated good performance, the most crucial aspect is that cocrystallization requires a matching coformer substance that can form a reliably strong intermolecular interaction, such as a hydrogen bond or stacking/T-type interaction,^33^ with the target compound. Consequently, cocrystal screening is unavoidable if a suitable coformer has not been discovered yet. Besides experimental methods, computational means such as virtual screening or crystal structure prediction are other possible ways to rapidly expand the coformer library.^34,35^ In case there are multiple coformers, the most suitable candidate should be the one that can swing the equilibrium toward the highest product purity with the least quantity of itself and the solvent. Another issue is that some coformers may not specifically bind to the target but also cocrystallize with the impurity at the same time. If this is the case, purification should still be feasible by making use of the difference in the coformer affinity between the product and its impurities,^21,22^ albeit it may be more complicated as the experimenter may need to construct and consider the phase diagram of several cocrystals at the same time. Additionally, solvent selection also plays a crucial role in determining the congruency of the ternary system.^31^ As shown in this work, an incongruent system is more preferable for separation. Initial screening is thus unavoidable, which, at its most basic, can be done by suspending the cocrystal in a selected solvent. If the cocrystal stoichiometric ratio of the residual solids is maintained, it is congruent; otherwise, it is incongruent. Last, one should realize that the cocrystal phase diagram is made by using pure components without taking impurity species into account, while the actual purification process is done in the presence of impurities. As a consequence, there is a limit beyond which the phase diagram is not applicable. In this study, that threshold was ∼70 wt % CUR purity; lower than this value risked complete dissolution.

Nonetheless, given that some of the plant-derived bioactive compounds can form cocrystals, such as caffeine,^36^ quercetin,^21,37^ myricetin,^21^ hesperitin,^38^ baicalein,^21,39^ chrysin,^40^ vanillin,^20^ and 11-azaartemisinin (a derivative of artemisinin),^41^ both cocrystallization and “coformer additive” methods presented in this study can be considered as additional options for purification. Similar to curcumin/demethoxycurcumin/bis­(demethoxy)­curcumin, the presented methods should also work even for a mixture containing chemically similar compounds, such as theobromine/theophylline/caffeine or baicalein/chrysin/galangin, as long as a suitable coformer is found.

## Supplementary Material



## Notes

(1) Preparation of pure curcumin standard, (2) preparation of α-resorcinol, (3) determination of curcumin stability upon drying, (4) determination of cocrystallization equilibrium time, (5) solubility measurement of purchased CUR in different EtOH:water ratio, (6) DSC scans for the CUR:RES binary phase diagram, (7) equilibrium data points of the CUR:RES:EtOH ternary phase diagram, (8) experiment data of curcumin extraction and purification from turmeric powders, (9) experiment data of resorcinol recycling, (10) ^1^H and ^13^C NMR spectra of recycled resorcinol, and (11) HPLC chromatograms of CUR solids produced in the “RES additive” experiment (PDF)
