# Supplementary material for: Phase Diagram-Enabled Curcumin Isolation and Purification by Utilizing Resorcinol as Cocrystal Former and Additive
Source: ACS Omega. 2026 Mar 17;11(12):19233–45. doi: 10.1021/acsomega.5c12529 (PMC13044614; doi:10.1021/acsomega.5c12529)
Supplement: Supplementary file 1 [file ao5c12529_si_001.pdf]

Phase Diagram-Enabled Curcumin Isolation and Purification by Utilizing Resorcinol as Co-Crystal Former and Additive

Yu-Rong Weng,▲ Ya-Hsuan Huang,▲ Jhe-Wei Wu, Dhanang Edy Pratama,* and Tu Lee

Department of Chemical and Materials Engineering, National Central University,

300 Zhongda Road, Zhongli District, Taoyuan City 320317, Taiwan (R.O.C.)

▲ These authors contributed equally

* Corresponding Author. Tel: +886-3-4227151 ext. 27256. E-mail: edypratama@ncu.edu.tw

PREPARATION OF PURE CURCUMIN STANDARD

Form I curcumin standard was prepared by performing cooling crystallization three times using a positive azeotrope composed of 1:44:55 (w/w/w) water:ethanol:acetonitrile (AZE-water/EtOH/ACN).[1] Purchased curcumin was dissolved in AZE-water/EtOH/ACN according to its solubility in the azeotrope at 60 °C of 58.8 mg/mL. The solution was first heated to 65 °C to ensure complete dissolution, then cooled to 25 °C in a water bath at a rate of 10 °C/min and held at 25 °C for 24 h. In each crystallization process, 1 wt% of curcumin seed crystals with a purity of 98 wt% that was prepared following the procedure of Tseng et al.,[2] was added to the solution based on the initial mass of curcumin when the temperature reached 50 °C. The seeded suspension was then aged at 25 °C for 24 h to complete crystallization. The resulting crystals were filtered using 1 μm pore size filter paper and dried in an oven at 60 °C for 24 h. This crystallization process was repeated three times. The final purified product with the final purity of 98.2 wt%, as determined by high-performance liquid chromatography (HPLC), was used as the curcumin standard for the construction of the phase diagram. In this study, Form I curcumin was exclusively used throughout our experimental procedures. Unless otherwise specified, any mention of curcumin or CUR throughout this work refers specifically to Form I.

PREPARATION OF α-RESORCINOL

To ensure the consistency of the resorcinol polymorph, the preparation of α-form resorcinol was carried out through three temperature cycling steps. Based on its measured solubility in water at 60°C, as determined by the gravimetric titration method[3] of 3466.67 mg/mL, 1.5 times the amount of resorcinol relative to this particular solubility value was partially dissolved in water and heated to 65°C at a rate of 1°C/min. After 65 °C was reached, the solution was aged for 80 min, then cooled to 25°C at a rate of 5°C/h and held for 14 h. The heating-cooling cycle was repeated thrice, with the temperature-history profile is shown in Figure S1.

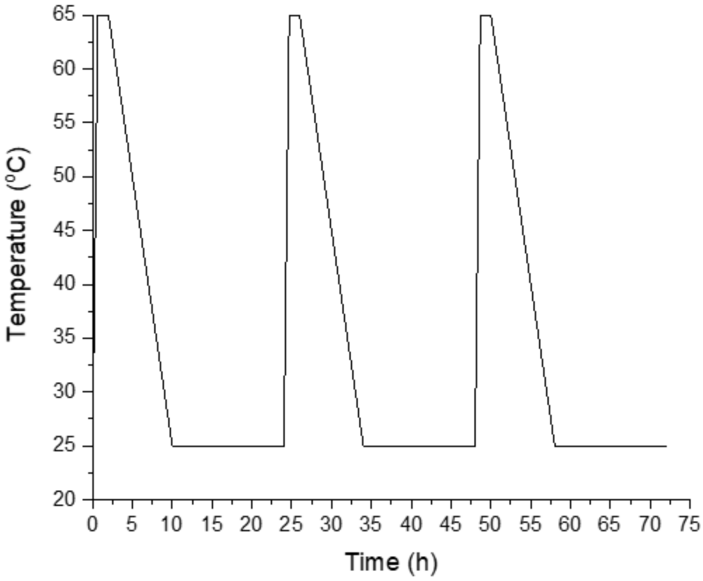

Figure S1. Temperature-history profile of the temperature cycling process used to prepare α-form resorcinol.

Figure S2 shows the PXRD patterns of the purchased resorcinol (Figure S2(a)) and the resorcinol after temperature cycling (Figure S2(b)). Both patterns match the characteristic 2θ peaks of α-resorcinol. In the DSC scans of the purchased resorcinol in Figure S3(a), the first endothermic peak, observed at approximately 106.5 °C, is attributed to the solid-solid transition of α- to β-resorcinol. Subsequently, β-resorcinol melts at 111.5 °C, as indicated by the second sharp endothermic peak. This is close to the value of 112.5 °C as observed by Ossowska-Chruściel et al.[4] Unlike the initial sample, the post-temperature cycle samples in Figure S3(b) only showed a single endothermic peak at approximately 112°C. We hypothesized that repeated thermal treatment leads to the formation of a more perfect crystal lattice with tighter molecular packing and better crystallinity. As a result, the α to β transition became negligible upon heating, and the melting point of α-resorcinol may have increased and approached that of the β-form, causing the two individual melting peaks to converge into one. Thus, we speculate that resorcinol exists as an enantiotropic polymorphic system, with the α-form being the thermodynamically stable phase at ambient conditions, while the β-form becomes more stable at elevated temperatures. By taking into account these results, temperature cycling was still conducted to further ensure polymorphic consistency, especially considering the potential of undetected traces of other polymorphs due to the qualitative nature of PXRD and DSC.

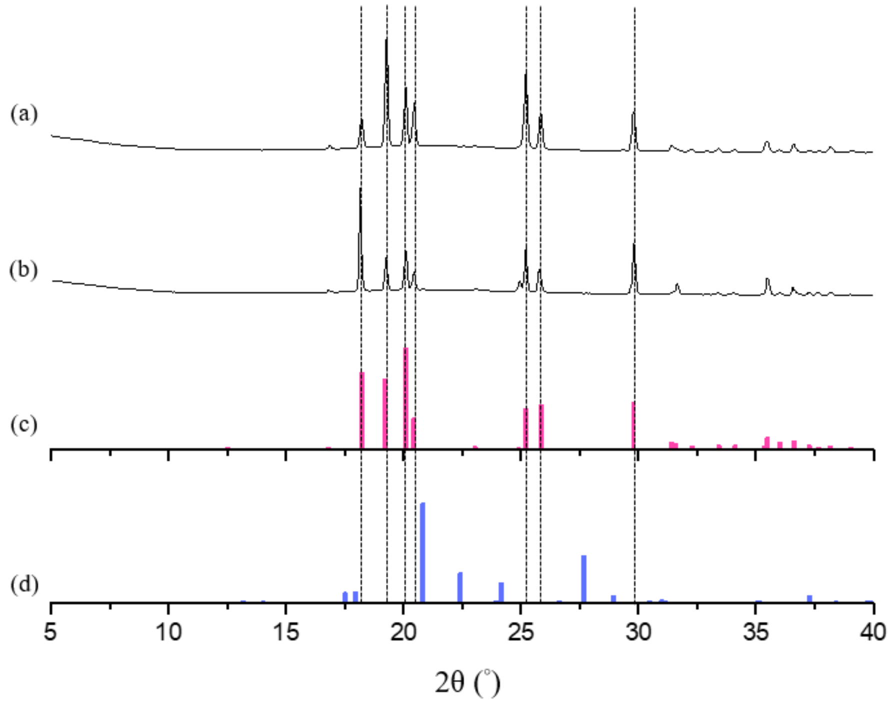

Figure S2. PXRD pattern of (a) the purchased resorcinol, (b) the purchased resorcinol after three temperature cycles, (c) α-resorcinol, and (d) β-resorcinol simulated PXRD pattern standards from CCDC (deposition numbers: 1248705,[5] and 1913240,[6] respectively.)

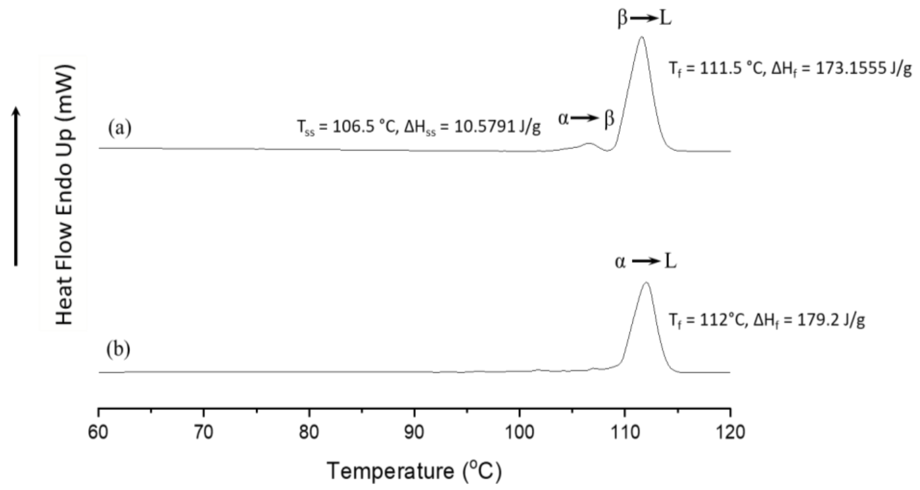

Figure S3. DSC scans of (a) the purchased resorcinol, and (b) the purchased resorcinol after three temperature cycle. T_{ss} and ΔH_{ss} denote solid-solid transformation temperature and enthalpy of α- to β-resorcinol, respectively. T_f and ΔH_f denote fusion temperature and enthalpy, respectively.

CURCUMIN STABILITY UPON DRYING

Since a CUR species is known to be prone to self-oxidation, a test was carried out to check if drying CUR solids at 60°C would cause any chemical change. Firstly, solids of CUR standard (~98.2 wt% purity) were placed in an oven, and the temperature was set at 60°C. Powder samples were taken at 24 h, 48 h, and 72 h. ^{1}H NMR spectroscopy was carried out to determine its chemical integrity. As shown in the NMR spectra in Figure S4, no change was observed. This result corroborates another research finding, which revealed that CUR undergoes thermal degradation starting from ~470 K (~196.85°C).[7]

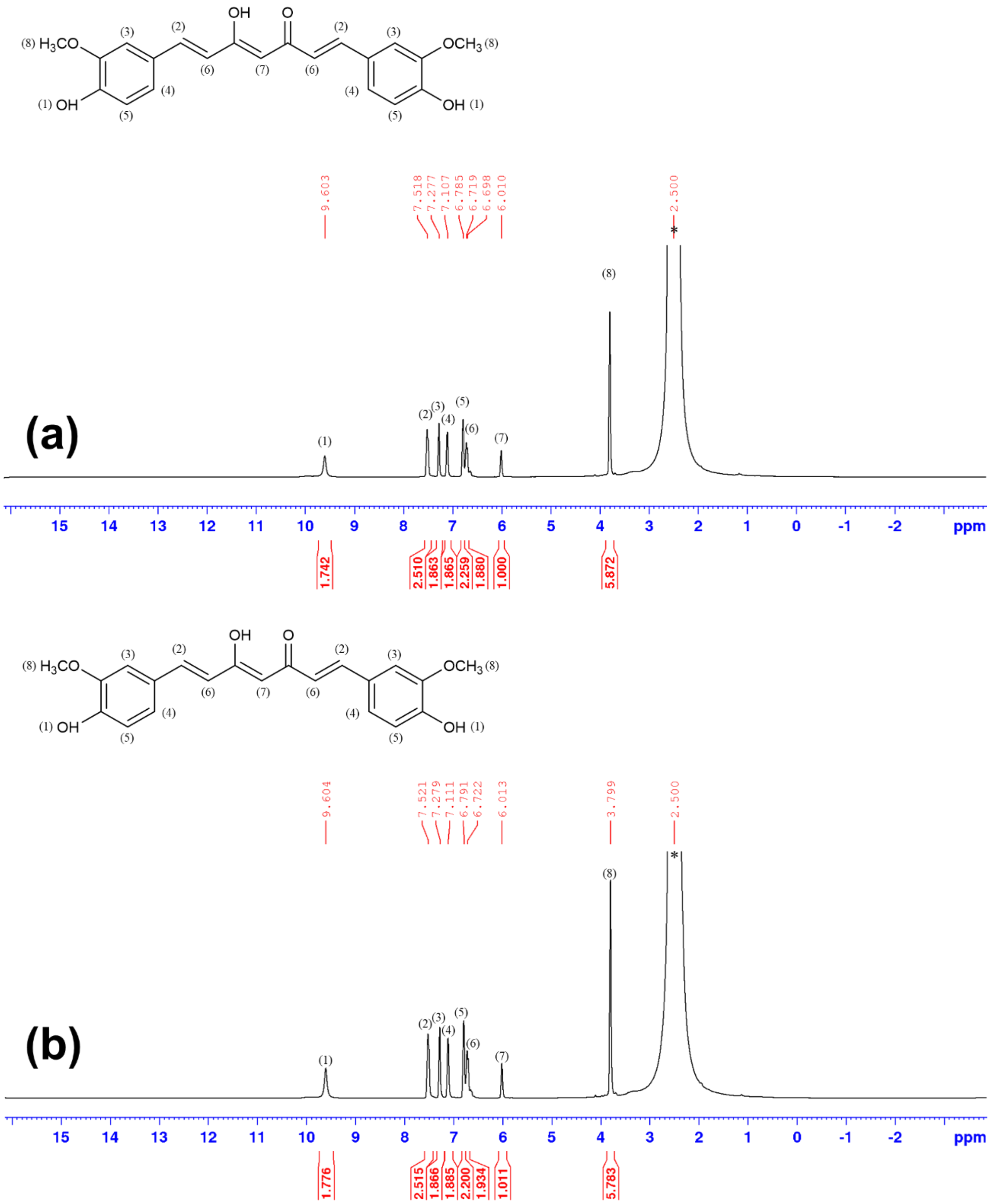

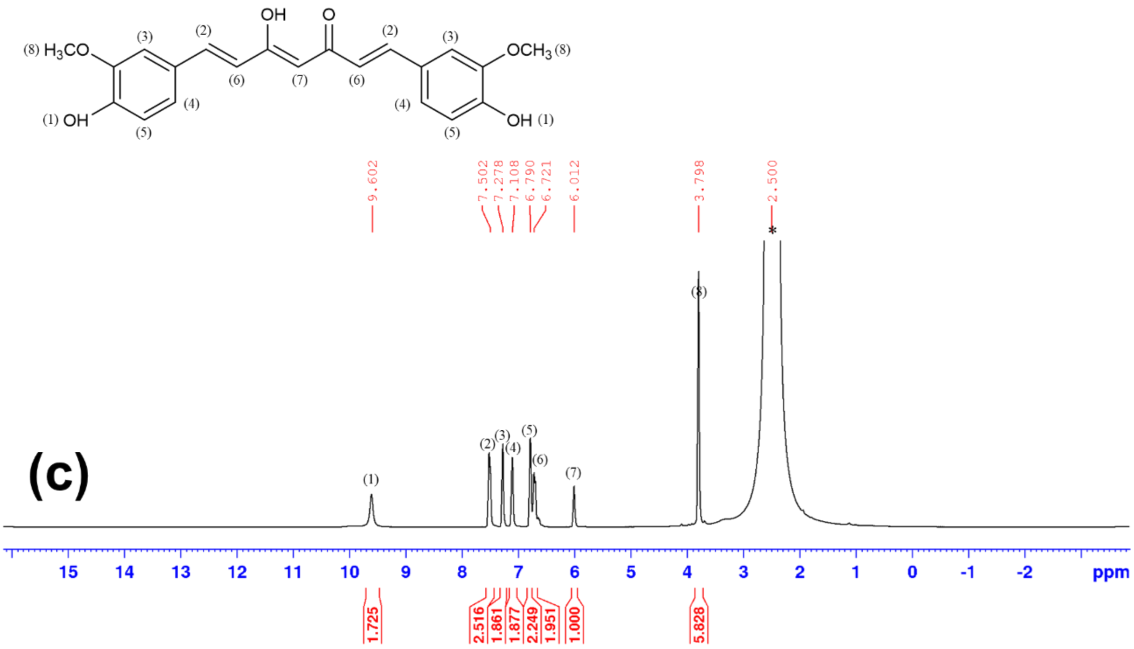

Figure S4. ^{1}H NMR spectrum of CUR standard after being subjected to thermal annealing at 60°C for (a) 24 h, (b) 48 h, and (c) 72 h.

DETERMINATION OF CO-CRYSTALLIZATION EQUILIBRIUM TIME

In order to ensure a proper thermodynamic equilibrium during co-crystallization, the following preliminary experiment was carried out. Purchased CUR (~70% purity), RES, and EtOH were mixed with the RES:CUR mole ratio set at 9:1 and a Q value of 7. The suspension was allowed to stand in a water bath at 25°C. Aliquots were withdrawn at different time intervals, each filtered by using a 0.22 µm PVDF hydrophilic syringe filter, and placed in an oven at 60°C until complete dryness. The mass ratio of the residual solids with the evaporated EtOH, i.e., the inverse of the Q value, was calculated based on the mass differences before and after drying. This experiment was repeated for standard CUR material (~98% purity). As shown in Figure S5, the one with lower CUR purity could reach equilibrium faster (2 h) than the one with higher CUR purity (24 h), possibly due to the higher solubility of the former. Henceforth, the co-crystallization time in this work was set at 24 h regardless of the initial CUR purity to ensure proper equilibrium attainment.

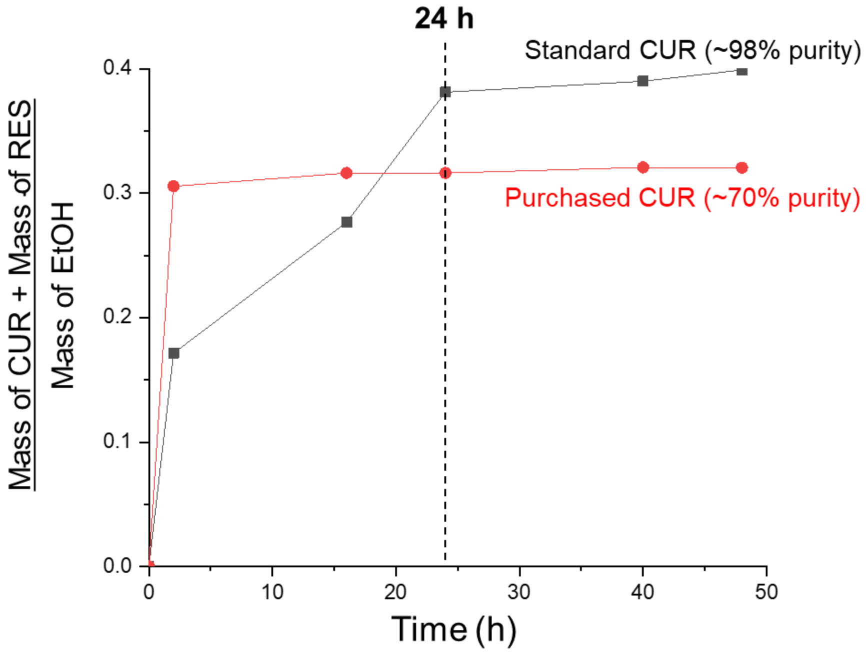

Figure S5. Time evolution of the mother liquor solute-to-solvent ratio of the purchased CUR (red circles) and standard CUR (black squares) during its co-crystallization with RES at 25°C.

SOLUBILITY MEASUREMENT OF PURCHASED CUR IN DIFFERENT ETHANOL:WATER RATIO

To evaluate the effect of water addition on the solubility of curcuminoids, solubility tests were conducted using EtOH-water mixtures at water volume fractions of 0%, 25%, 50%, 75%, and 100%. For each condition, 5 mg of purchased CUR (70 wt% purity) was accurately weighed into 20 mL, 100 mL, and 200 mL vials. The samples were placed in a 25 °C water bath, and the designated solvent mixture was slowly added until complete dissolution occurred. The required volume was recorded to calculate the solubility value. The plot of purchased CUR solubility values at different EtOH-water volume ratios is shown in Figure S6. The inflection point, where the slope starts to become narrower, is located at 50% water volume fraction, or 1:1 (v/v) EtOH-water ratio. Therefore, this value was deemed to be the appropriate amount of water to be added if one wants to efficiently precipitate out curcuminoids.

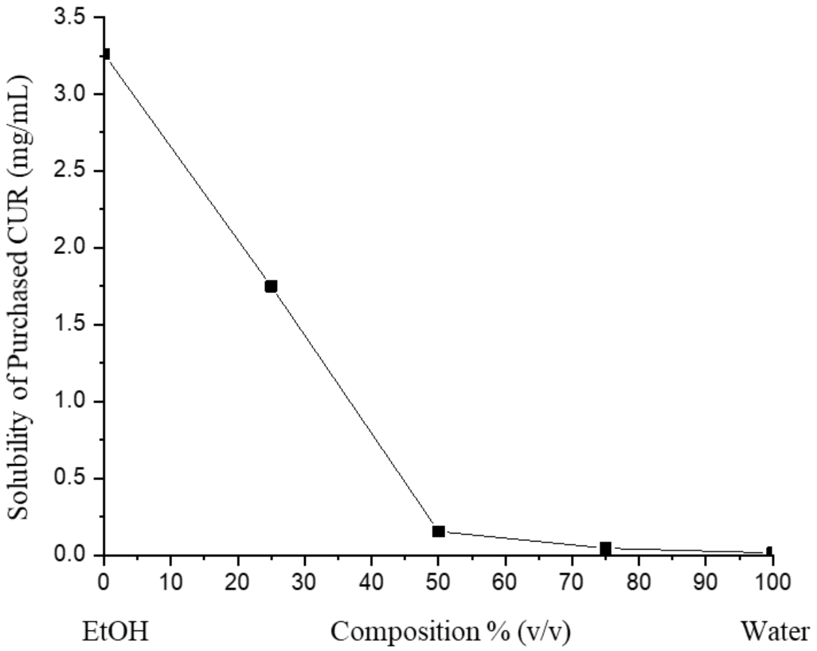

Figure S6. Solubility of purchased CUR in EtOH-water mixtures with different volume ratios at 25 °C and ambient pressure.

DSC SCANS FOR CUR:RES BINARY PHASE DIAGRAM

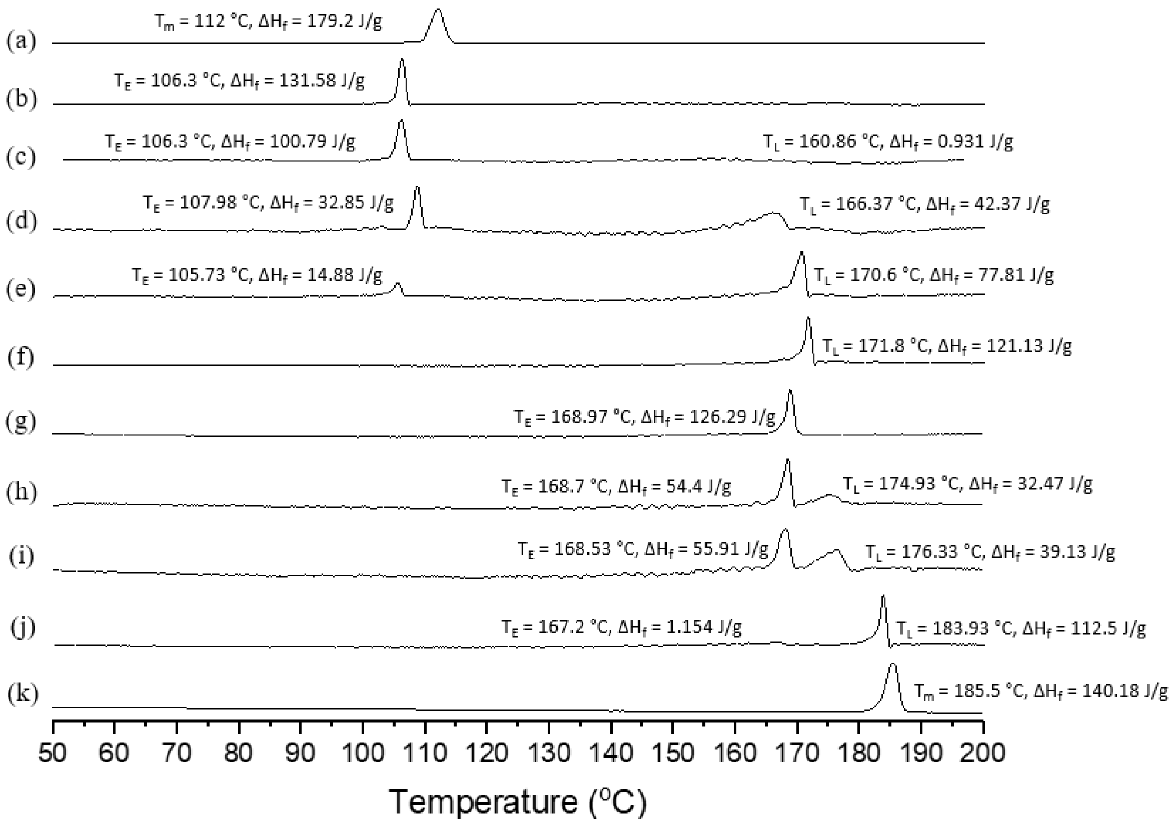

Figure S7. DSC scans of the CUR–RES binary mixtures used to construct the T-x binary phase diagram. Samples (a) to (k) correspond to mixtures ranging from 0% CUR + 100% RES to 100% CUR + 0% RES, with mole fraction of curcumin increasing in increments of 10 mol%. The peak temperature and associated enthalpy change are labeled for each sample. These thermal events were used to identify eutectic points and the co-crystal melting behavior, and subsequently to construct the binary phase diagram presented in Figure 5. T_m: melting temperature, T_L: liquidus temperature, T_E: eutectic temperature, and ΔH_f: enthalpy of fusion.

EQUILIBRIUM DATA POINTS OF CUR:RES:EtOH TERNARY PHASE DIAGRAM

Table S1. Summary of each data point of tie-lines from solid-solid-liquid CUR-RES-EtOH ternary phase diagram in Figure 6, including solid-liquid equilibrium of pure component of (i) CUR, and (j) RES.

	M		I		R		M′		R′	
	Q	x_{CUR}	Q	x_{CUR}	Q	x_{CUR}	Q	x_{CUR}	Q	x_{CUR}
(a)	104.88	0.058	79.9	0.199	30.65	0.98	98.27	0.058	0	1
(b)	45.69	0.026	40.12	0.2	0.85	14.64	46.18	0.026	0	0.95
(c)	19.28	0.0143	9.78	0.5	4.54	0.78	19.24	0.0143	0	0.8
(d)	18.54	0.012	19.98	0.1	11	0.69	19.72	0.012	0	0.7
(e)	13.33	0.01	15	0.1	8.23	0.45	14.63	0.01	0	0.5
(f)	8.19	0.0068	10.03	0.1	4.73	0.5	9.385	0.0068	0	0.5
(g)	6.08	0.0059	7.02	0.1	3.64	0.445	6.77	0.0059	0	0.5
(h)	5.74	0.0058	4.9	0.1	3.095	0.315	5.73	0.0058	0	0.4
(i)	1703.73	1	984.96	1	107.72	1	N/A	N/A	N/A	N/A
(j)	1.39	0	0.89	0	0.26	0	N/A	N/A	N/A	N/A

$Q = \frac{Mole\ of\ EtOH}{Mole\ of\ CUR + Mole\ of\ RES}$

x_{CUR} = Mole fraction of CUR = $\frac{Mole\ of\ CUR}{Mole\ of\ CUR + Mole\ of\ RES}$

EXPERIMENT DATA OF CURCUMIN EXTRACTION AND PURIFICATION FROM TURMERIC POWDERS

Table S2. Data from the first run of the three repeated curcumin purification experiment. CUR samples labeled with letters in bracketed parentheses are the ones characterized with HPLC, whose chromatograms are shown in Figure S8.

Stage	Input	Output	Q value	CUR purity (wt%)	Yield (wt%)
Turmeric extraction	• Turmeric (100 g)<br>• EtOH (200 mL)	• Oleoresin (5.834 g) **[A]**	N/A[a]	22.037	5.83
Solid-liquid equilibrium	• Oleoresin (5.834 g)<br>• EtOH (18.46 mL)	• Crude CUR (0.949 g) **[B]**	N/A[a]	75.38	16.27
Co-crystal formation #1	• Crude CUR (0.949 g, equivalent to 0.00194 mol pure CUR)<br>• RES (1.924 g, 0.0174 mol)<br>• EtOH (7.924 mL, 0.1359 mol)	• Co-crystal #1 (0.804 g, 0.00168 mol)	7	N/A[b]	N/A[b]
Co-crystal dissociation #1	• Co-crystal #1 (0.804 g, 0.00168 mol)<br>• EtOH (24.491 mL, 0.42 mol)	• Purified CUR powders #1 (0.3972 g, equivalent to 0.00095 mol pure CUR) **[C]**	125	88.21	49.4
Co-crystal formation #2	• Purified CUR powders #1 (0.3972 g, equivalent to 0.00095 mol pure CUR)<br>• RES (0.942 g, 0.00855 mol)<br>• EtOH (3.879 mL, 0.0665 mol)	• Co-crystal #2 (0.3883 g, 0.00081 mol)	7	N/A[b]	N/A[b]
Co-crystal dissociation #2	• Co-crystal #2 (0.3883 g, 0.00081 mol)<br>• EtOH (11.829 mL, 0.2028 mol)	• Purified CUR powders #2 (0.2846 g, equivalent to 0.00074 mol pure CUR) **[D]**	125	96.05	73.3

[a] The ternary phase diagram is inapplicable for turmeric extraction and solid-liquid equilibrium process.
[b] As the co-crystal serves only as an intermediate and not the final product, the purity and yield of curcumin are not addressed.

Yield (wt%) = $\frac{Output\ solids(g)}{Input\ solids(g)} \times 100\%$

Q = $\frac{Mole\ of\ EtOH}{Mole\ of\ CUR + Mole\ of\ RES}$

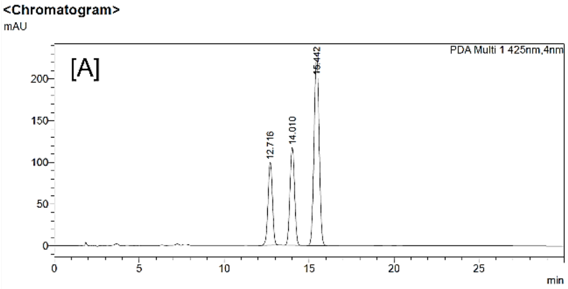

Name	Retention (min)	Peak Area	Peak Area Percentage (%)
BDMC	12.716	1781881	20.41
DMC	14.010	2218800	25.415
CUR	15.442	4729594	54.175

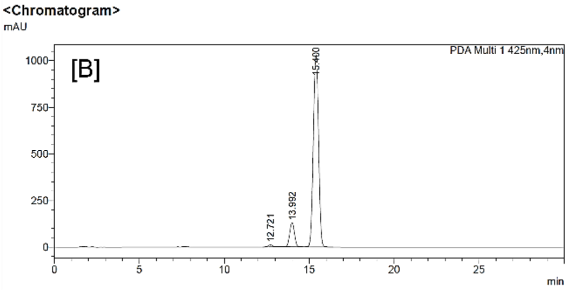

Name	Retention (min)	Peak Area	Peak Area Percentage (%)
BDMC	12.721	837840	3.913
DMC	13.992	2997616	14.002
CUR	15.400	1753551	82.085

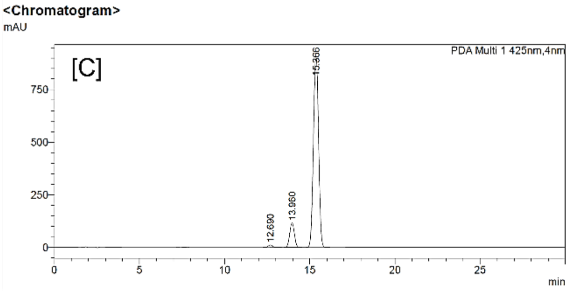

Name	Retention (min)	Peak Area	Peak Area Percentage (%)
BDMC	12.690	132456	0.592
DMC	13.960	2407229	10.758
CUR	15.366	19837390	88.651

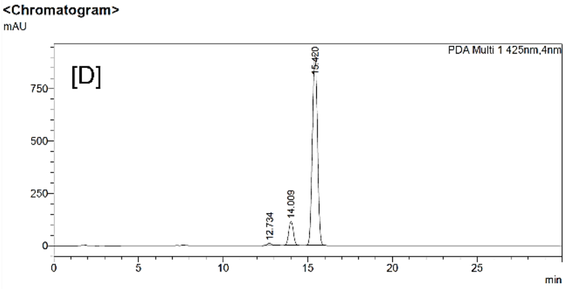

Name	Retention (min)	Peak Area	Peak Area Percentage (%)
BDMC	12.734	80506	0.407
DMC	14.009	1706559	8.634
CUR	15.420	17978335	90.959

Figure S8. HPLC chromatograms of CUR samples. The labels in each figure correspond to the CUR sample with the identical label in Table S2.

Table S3. Data from the second run of the three repeated curcumin purification experiment. CUR samples labeled with letters in bracketed parentheses are the ones characterized with HPLC, whose chromatograms are shown in Figure S9.

**Stage**	**Input**	**Output**	**Q value**	**CUR purity (wt%)**	**Yield (wt%)**
Turmeric extraction	• Turmeric (100 g)<br>• EtOH (200 mL)	• Oleoresin (5.23 g) **[E]**	N/A[a]	21.37	5.23
Solid-liquid equilibrium	• Oleoresin (5.23 g)<br>• EtOH (16.55 mL)	• Crude CUR (0.918 g) **[F]**	N/A[a]	75.56	17.56
Co-crystal formation #1	• Crude CUR (0.918 g, equivalent to 0.00188 mol pure CUR)<br>• RES (1.86 g, 0.0168 mol)<br>• EtOH (7.663 mL, 0.1314 mol)	• Co-crystal #1 (0.828 g, 0.00173 mol)	7	N/A[b]	N/A[b]
Co-crystal dissociation #1	• Co-crystal #1 (0.828 g, 0.00173 mol)<br>• EtOH (25.22 mL, 0.4325 mol)	• Purified CUR powders #1 (0.452 g, equivalent to 0.00113 mol pure CUR) **[G]**	125	92.765	54.6
Co-crystal formation #2	• Purified CUR powders #1 (0.452 g, equivalent to 0.00113 mol pure CUR)<br>• RES (1.128 g, 0.0102 mol)<br>• EtOH (4.645 mL, 0.0796 mol)	• Co-crystal #2 (0.403 g, 0.000842 mol)	7	N/A[b]	N/A[b]
Co-crystal dissociation #2	• Co-crystal #2 (0.403 g, 0.000842 mol)<br>• EtOH (12.27 mL, 0.21 mol)	• Purified CUR powders #2 (0.269 g, equivalent to 0.00071 mol pure CUR) **[H]**	125	97.26	66.8

[a] The ternary phase diagram is inapplicable for turmeric extraction and solid-liquid equilibrium process.
[b] As the co-crystal serves only as an intermediate and not the final product, the purity and yield of curcumin are not addressed.

Yield (wt%) = $\frac{Output\ solids(g)}{Input\ solids(g)} \times 100\%$

Q = $\frac{Mole\ of\ EtOH}{Mole\ of\ CUR + Mole\ of\ RES}$

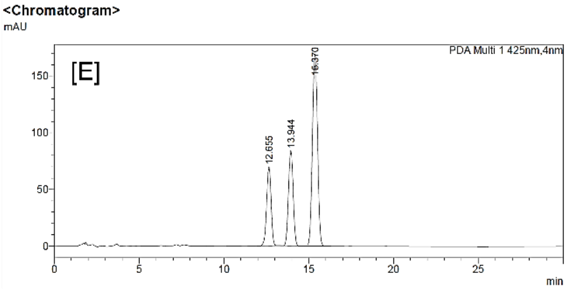

Name	Retention (min)	Peak Area	Peak Area Percentage (%)
BDMC	12.665	1522198	18.392
DMC	13.944	2039106	24.637
CUR	15.370	4715249	56.971

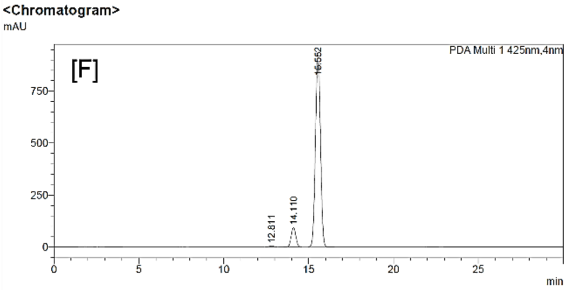

Name	Retention (min)	Peak Area	Peak Area Percentage (%)
BDMC	12.811	25482	2.365
DMC	14.110	3150710	13.8391
CUR	15.552	19077596	83.796

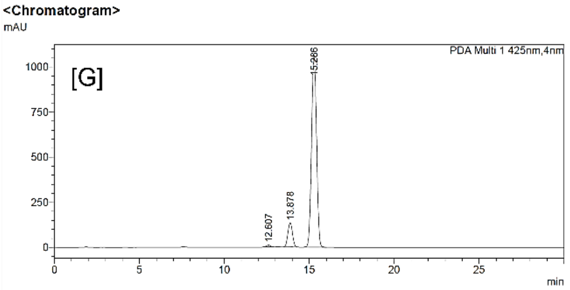

Name	Retention (min)	Peak Area	Peak Area Percentage (%)
BDMC	12.607	178239	0.828
DMC	13.878	2638916	12.266
CUR	15.286	18697582	86.906

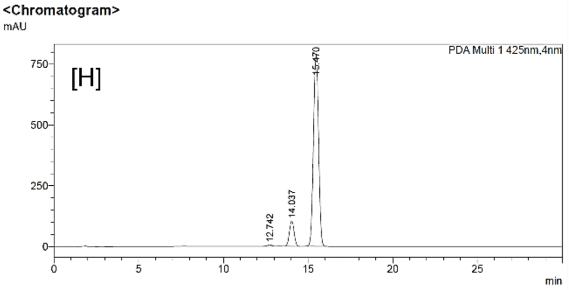

Name	Retention (min)	Peak Area	Peak Area Percentage (%)
BDMC	12.742	20971	1.768
DMC	14.037	2401827	10.608
CUR	15.470	19839328	87.624

Figure S9. HPLC chromatograms of CUR samples. The labels in each figure correspond to the CUR sample with the identical label in Table S3.

Table S4. Data from the third run of the three repeated curcumin purification experiment. CUR samples labeled with letters in bracketed parentheses are the ones characterized with HPLC, whose chromatograms are shown in Figure S10.

Stage	Input	Output	Q value	CUR purity (wt%)	Yield (wt%)
Turmeric extraction	• Turmeric (100 g)<br>• EtOH (200 mL)	• Oleoresin (6 g) **[I]**	N/A[a]	20.38	6
Solid-liquid equilibrium	• Oleoresin (6 g)<br>• EtOH (18.98 mL)	• Crude CUR (0.876 g) **[J]**	N/A[a]	73.28	14.6
Co-crystal formation #1	• Crude CUR (0.876 g, equivalent to 0.00174 mol pure CUR)<br>• RES (1.726 g, 0.0156 mol)<br>• EtOH (7.109 mL, 0.122 mol)	• Co-crystal #1 (0.765 g, 0.001598 mol)	7	N/A[b]	N/A[b]
Co-crystal dissociation #1	• Co-crystal #1 (0.765 g, 0.001598 mol)<br>• EtOH (23.32 mL, 0.3999 mol)	• Purified CUR powders #1 (0.37 g, equivalent to 0.000876 mol pure CUR **[K]**	125	87.25	48.98
Co-crystal formation #2	• Purified CUR powders #1 (0.37 g, equivalent to 0.000876 mol pure CUR<br>• RES (0.868 g, 0.00788 mol)<br>• EtOH (3.575 mL, 0.0613 mol)	• Co-crystal #2 (0.32 g, 0.000668 mol)	7	N/A[b]	N/A[b]
Co-crystal dissociation #2	• Co-crystal #2 (0.32 g, 0.000668 mol)<br>• EtOH (9.748 mL, 0.167 mol)	• Purified CUR powders #2 (0.226 g, equivalent to 0.000582 mol pure CUR) **[L]**	125	94.87	70.6

[a] The ternary phase diagram is inapplicable for turmeric extraction and solid-liquid equilibrium process.
[b] As the co-crystal serves only as an intermediate and not the final product, the purity and yield of curcumin are not addressed.

Yield (wt%) = $\frac{Output\ solids(g)}{Input\ solids(g)} \times 100\%$

Q = $\frac{Mole\ of\ EtOH}{Mole\ of\ CUR + Mole\ of\ RES}$

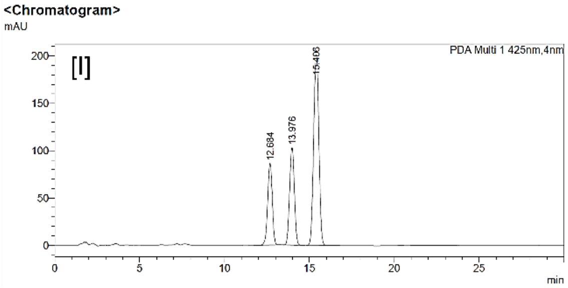

Name	Retention (min)	Peak Area	Peak Area Percentage (%)
BDMC	12.684	1522198	18.392
DMC	13.976	2039106	24.637
CUR	15.406	4715249	56.971

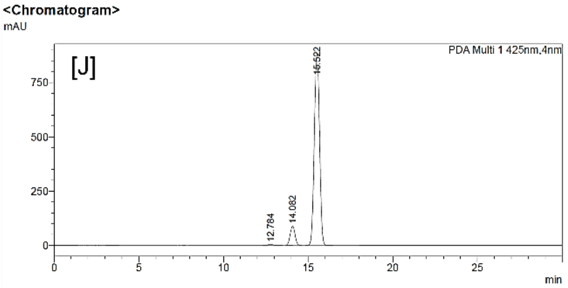

Name	Retention (min)	Peak Area	Peak Area Percentage (%)
BDMC	12.784	183291	1.057
DMC	14.082	2100277	12.112
CUR	15.522	15057288	86.831

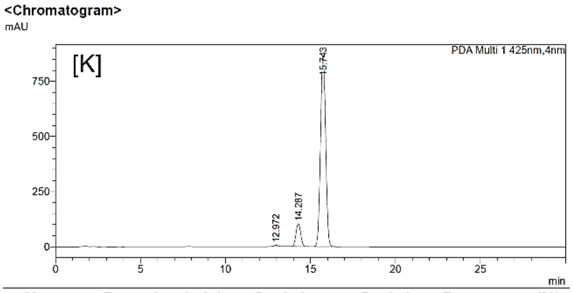

Name	Retention (min)	Peak Area	Peak Area Percentage (%)
BDMC	12.972	84474	0.424
DMC	14.287	2078207	10.442
CUR	15.743	17738803	89.133

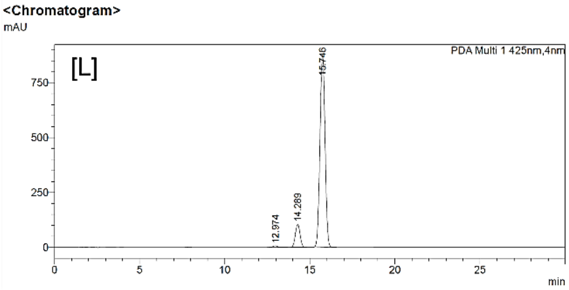

Name	Retention (min)	Peak Area	Peak Area Percentage (%)
BDMC	12.974	42614	0.218
DMC	14.289	1347360	6.881
CUR	15.746	18191707	92.902

Figure S10. HPLC chromatograms of CUR samples. The labels in each figure correspond to the CUR sample with the identical label in Table S4.

EXPERIMENT DATA OF RESORCINOL RECYCLING

Table S5. Design of the first stage cocrystallization purification process for curcumin. CUR samples labeled with letters in bracketed parentheses are the ones characterized with HPLC, whose chromatograms are shown in Figure S11. Note that the initial CUR materials labeled [M] in Runs #1, #2, and #3 are identical.

**Cocrystal Formation**					
**Number of run**	**Initial CUR purity (wt%)**	**Curcumin (mg)**	**Resorcinol (mg)**	**Solvent (mL)**	**Product (mg) (Cocrystal)**
#1	70.0 **[M]**	5000.0	9414.0	38.8	4722.1
#2	70.0 **[M]**	5000.2	9413.7	38.8	4861.5
#3	70.0 **[M]**	5000.4	9414.3	38.8	4881.8
Average	70.0 ± 0.00	5000.2 ± 0.12	9414.0 ± 0.17	38.8 ± 0.00	4821.8 ± 49.9
**Cocrystal Dissociation**					
**Number of run**	**Cocrystal (mg)**	**Solvent (mL)**	**Product (mg) (Purified CUR)**	**Yield* (wt%)**	**Purity (wt%)**
#1	4722.1	143.8	3331.5 **[N]**	66.6	88.0
#2	4861.5	148.1	3115.5 **[O]**	62.3	94.0
#3	4881.8	148.7	3180.2 **[P]**	63.6	92.0
Average	4821.8 ± 49.9	146.9 ± 1.52	3209.1 ± 66.4	64.2 ± 1.30	91.3 ± 1.77
**Resorcinol Recovery and Recycling**					
**Number of run**	**Mother liquor (mL)**	**Product (mg) (Recycled RES)**	**Yield* (wt%)**	**Purity**	
#1	34.0	7385.4	78.4	Verified by NMR	
#2	31.0	7832.0	83.2	Verified by NMR	
#3	28.0	7551.5	80.2	Verified by NMR	
Average	31.0 ± 1.73	7589.6 ± 129.7	80.6 ± 1.40	Verified by NMR	

* The yield was calculated as the ratio of the weight of the final product to that of the raw material.

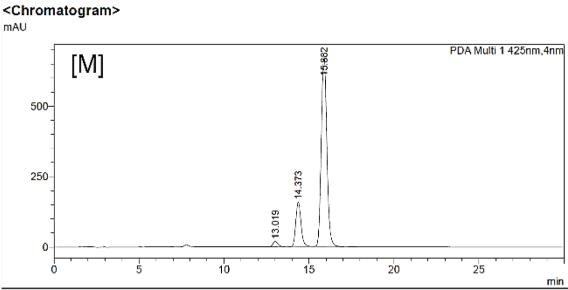

Name	Retention (min)	Peak Area	Peak Area Percentage (%)
BDMC	13.019	378245	1.98
DMC	14.373	3301950	17.287
CUR	15.882	15420946	80.733

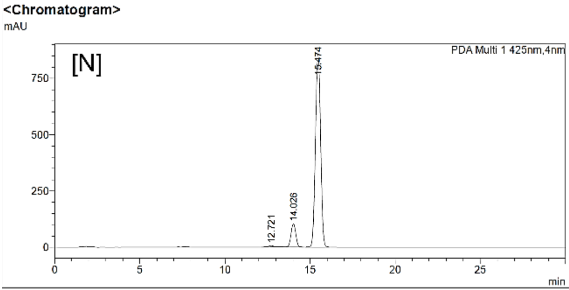

Name	Retention (min)	Peak Area	Peak Area Percentage (%)
BDMC	12.806	177149	0.789
DMC	14.111	2730148	12.291
CUR	15.561	19305295	86.911

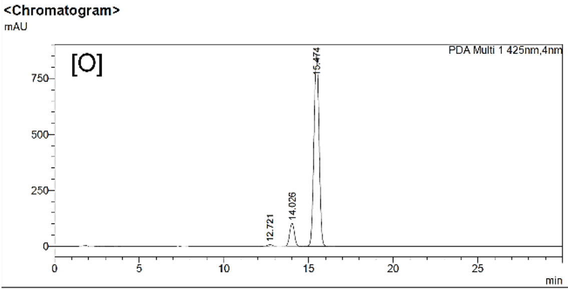

Name	Retention (min)	Peak Area	Peak Area Percentage (%)
BDMC	12.721	25618	1.757
DMC	14.026	2704896	10.608
CUR	15.474	118825981	87.635

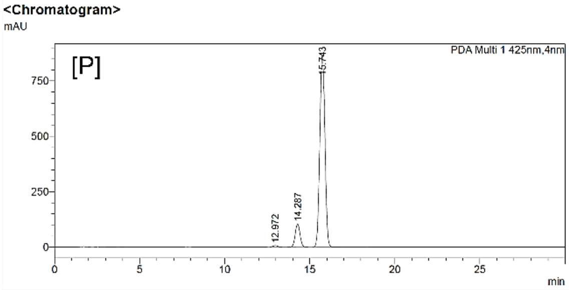

Name	Retention (min)	Peak Area	Peak Area Percentage (%)
BDMC	12.972	20837	1.808
DMC	14.287	2447381	10.572
CUR	15.743	20283865	87.620

Figure S11. HPLC chromatograms of CUR samples. The labels in each figure correspond to the CUR sample with the identical label in Table S5.

Table S6. Design of the second stage cocrystallization purification process for curcumin. CUR samples labeled with letters in bracketed parentheses are the ones characterized with HPLC, whose chromatograms are shown in Figure S12.

Cocrystal Formation						
**Number of run**	**Initial CUR purity (wt%)**	**Purified CUR (mg)**	**Resorcinol (mg)**	**Recycled RES (mg)**	**Solvent (mL)**	**Product(mg) (Cocrystal)**
#1	88.0	3331.5	500.6	7385.4	32.5	3409.9
#2	94.0	3115.5	45.5	7832.0	32.4	3556.3
#3	92.0	3180.2	318.5	7551.5	32.4	3475.6
Average	91.3 ± 1.77	3209.1 ± 66.4	288.2 ± 108.2	7589.6 ± 129.7	32.4 ± 0.06	3480.6 ± 42.3

Cocrystal Dissociation					
**Number of run**	**Cocrystal (mg)**	**Solvent (mL)**	**Product (mg) (Purified CUR)**	**Yield (wt%)**	**Purity (wt%)**
#1	3409.9	103.9	2389.0 **[Q]**	71.7	94.5
#2	3556.3	108.3	2192.6 **[R]**	70.4	98.2
#3	3475.6	105.9	2276.2 **[S]**	71.6	95.4
Average	3480.6 ± 42.3	106.0 ± 0.78	2285.9 ± 48.6	71.2 ± 0.42	96.0 ± 1.11

Resorcinol Recovery and Recycling				
**Number of run**	**Mother liquor (mL)**	**Product (mg) (Recycled RES)**	**Yield (wt%)**	**Purity**
#1	29	6436.1	81.6	Verified by NMR
#2	25	5782.5	73.4	Verified by NMR
#3	27	6229.4	79.2	Verified by NMR
Average	27 ± 1.15	6149.3 ± 152.6	78.1 ± 2.44	Verified by NMR

[*] The yield was calculated as the ratio of the weight of the final product to that of the raw material.

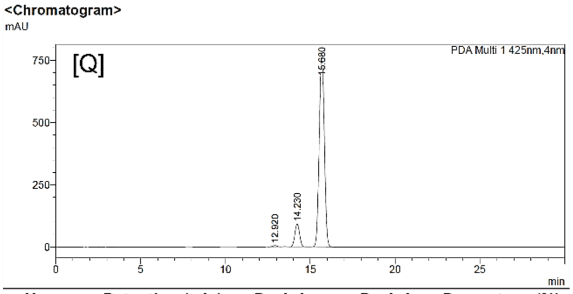

Name	Retention (min)	Peak Area	Peak Area Percentage (%)
BDMC	12.920	25618	2.204
DMC	14.230	2704896	12.296
CUR	15.680	118825981	85.58

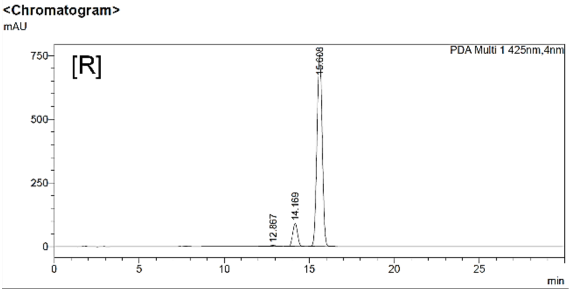

Name	Retention (min)	Peak Area	Peak Area Percentage (%)
BDMC	12.867	10175	1.317
DMC	14.169	456475	6.631
CUR	15.608	21543797	92.052

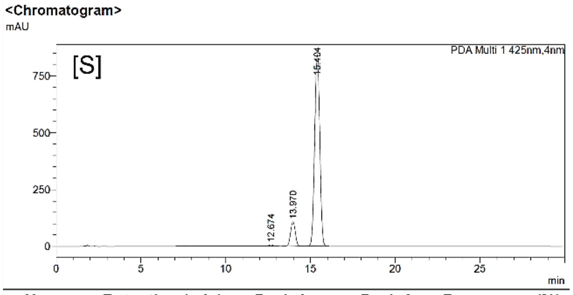

Name	Retention (min)	Peak Area	Peak Area Percentage (%)
BDMC	12.674	15804	1.317
DMC	13.970	1374658	6.631
CUR	15.404	19084361	92.052

Figure S12. HPLC chromatograms of CUR samples. The labels in each figure correspond to the CUR sample with the identical label in Table S6.

NMR SPECTRA OF RECYCLED RESORCINOL

NMR spectroscopy was employed to qualitatively assess the chemical purity of the recycled RES. In the ^{1}H NMR spectrum acquired in D_2O, resorcinol exhibited three characteristic chemical shifts at δ = 6.97, 6.33, and 6.28 ppm, corresponding to the three nonequivalent aromatic protons on the benzene ring. Additionally, a peak at δ = 4.80 ppm corresponds to the residual of D_2O,[8] as shown in Figure S13. The ^{13}C NMR spectrum displayed four distinct signals at δ = 156.69, 130.65, 107.52, and 102.56 ppm, representing the different carbon environments in the resorcinol molecule, as shown in Figure S14. These NMR results are consistent with the Spectral Database for Organic Compounds, SDBS No. 1501, for pure resorcinol. No additional peaks or interfering signals were observed, indicating that the recycled RES samples were pure.

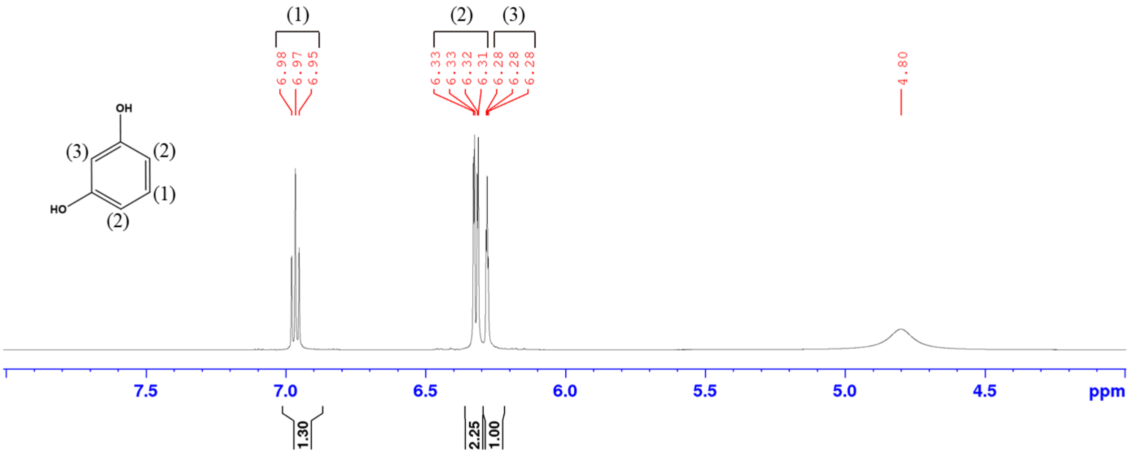

Figure S13. ^{1}H NMR spectrum of recycled RES in deuterium oxide.

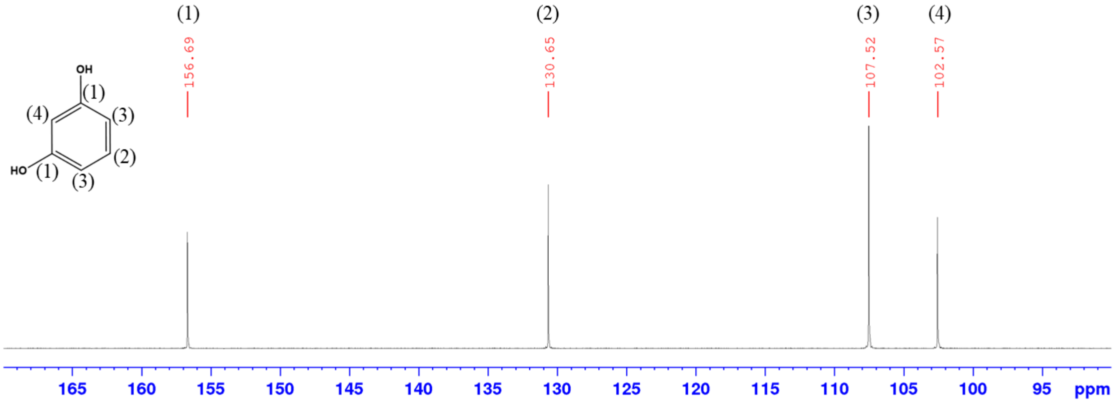

Figure S14. ^{13}C NMR spectrum of recycled RES in deuterium oxide.

HPLC CHROMATOGRAMS OF CUR SOLIDS PRODUCED IN “RES ADDITIVE” EXPERIMENTS

HPLC chromatograms of CUR solids produced in “RES Additive” experiments are shown in Figure S15 and Figure S16. Note that while the peak area ratios are more or less similar in both cases, the purity by assay (calibrated against a pure CUR standard) differed by a large margin, which are 85.3 ± 2.0 wt% and 78.5 ± 1.6 wt% with and without RES, respectively.

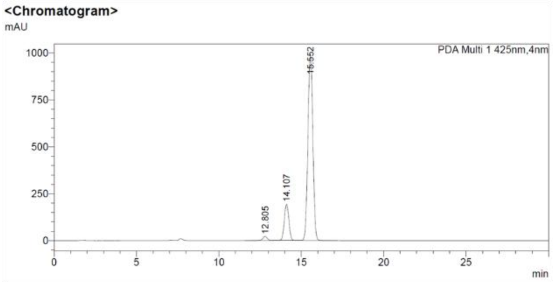

Name	Retention (min)	Peak Area	Peak Area Percentage (%)
BDMC	12.805	372018	1.504
DMC	14.107	3683608	14.895
CUR	15.552	20674400	83.600

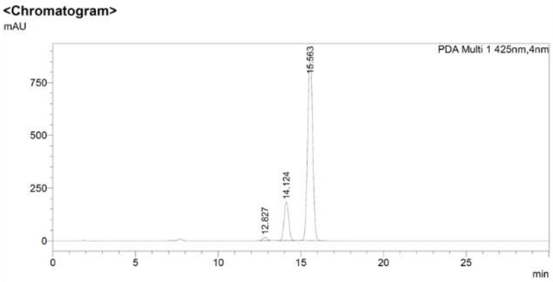

Name	Retention (min)	Peak Area	Peak Area Percentage (%)
BDMC	12.827	268713	1.197
DMC	14.124	3550374	15.819
CUR	15.563	18624003	82.983

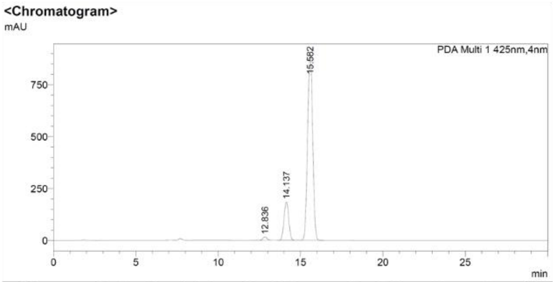

Name	Retention (min)	Peak Area	Peak Area Percentage (%)
BDMC	12.836	288560	1.244
DMC	14.137	3691898	15.912
CUR	15.582	119222159	82.845

Figure S15. HPLC chromatograms of triplicate experiments of CUR produced by suspending in EtOH in the presence of RES.

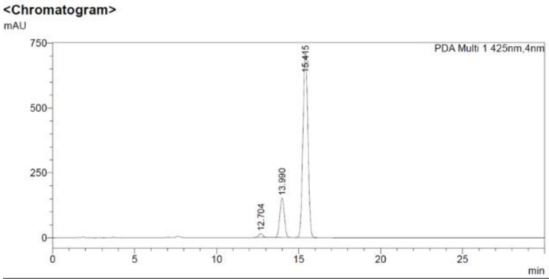

Name	Retention (min)	Peak Area	Peak Area Percentage (%)
BDMC	12.704	137937	0.992
DMC	13.990	2052526	14.756
CUR	15.415	11719368	84.252

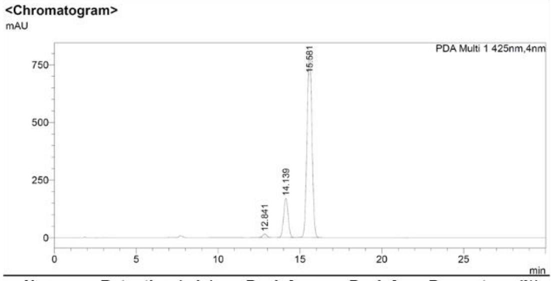

Name	Retention (min)	Peak Area	Peak Area Percentage (%)
BDMC	12.841	257828	1.269
DMC	14.139	3266424	16.081
CUR	15.581	16787799	82.649

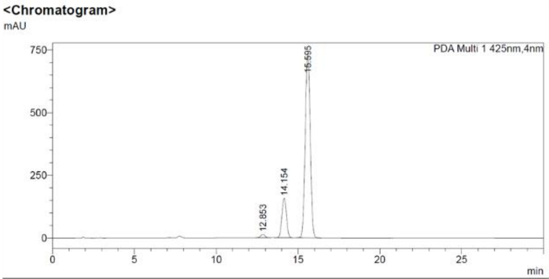

Name	Retention (min)	Peak Area	Peak Area Percentage (%)
BDMC	12.853	372018	1.504
DMC	14.154	3683608	14.895
CUR	15.595	20674400	83.600

Figure S16. HPLC chromatograms of triplicate experiments of CUR produced after suspending in EtOH without any RES.

REFERENCES

(1) Pratama, D. E.; Chen, Y.-H.; Lee, T. A Solid–Solid–Liquid Quasi-Ternary Phase Diagram Method for Multicomponent Solids Validated by Curcumin Purification Design. *Ind. Eng. Chem. Res.* **2024**, *63* (44), 19135–19152. https://doi.org/10.1021/acs.iecr.4c02142.

(2) Tseng, J. D.; Lee, H. L.; Yeh, K. L.; Lee, T. Recyclable Positive Azeotropes for the Purification of Curcumin with Optimum Purity and Solvent Capacity. *Chem. Eng. Res. Des.* **2022**, *180*, 200–211. https://doi.org/10.1016/j.cherd.2022.02.019.

(3) Lee, T.; Kuo, C. S.; Chen, Y. H. Solubility, Polymorphism, Crystallinity, and Crystal Habit of Acetaminophen and Ibuprofen by Initial Solvent Screening. *Pharm. Tech.* **2006**, *30* (10), 72–92.

(4) Ossowska-Chruściel, M. D.; Juszyńska-Gałązka, E.; Zając, W.; Rudzki, A.; Chruściel, J. Mesomorphic Properties of Resorcinol. *J. Mol. Struct.* **2015**, *1082*, 103–113. https://doi.org/10.1016/j.molstruc.2014.10.080.

(5) Robertson, J. M. The Space Group of Resorcinol $C_6H_6O_2$. *Z. Kristallogr. Cryst. Mater.* **1934**, *89* (1–6), 518. https://doi.org/10.1524/zkri.1934.89.1.518.

(6) Safari, F.; Olejniczak, A.; Katrusiak, A. Pressure-Dependent Crystallization Preference of Resorcinol Polymorphs. *Cryst. Growth Des.* **2019**, *19* (10), 5629–5635. https://doi.org/10.1021/acs.cgd.9b00610.

(7) Masih, R.; Iqbal, M. S. Thermal Degradation Kinetics and Pyrolysis GC–MS Study of Curcumin. *Food Chem.* **2022**, *385*, 132638. https://doi.org/10.1016/j.foodchem.2022.132638.

(8) Babij, N. R.; McCusker, E. O.; Whiteker, G. T.; Canturk, B.; Choy, N.; Creemer, L. C.; De Amicis, C. V.; Hewlett, N. M.; Johnson, P. L.; Knobelsdorf, J. A.; Li, F.; Lorsbach, B. A.; Nugent, B. M.; Ryan, S. J.; Smith, M. R.; Yang, Q. NMR Chemical Shifts of Trace Impurities: Industrially Preferred Solvents Used in Process and Green Chemistry. *Org. Process Res. Dev.* **2016**, *20* (3), 661–667. https://doi.org/10.1021/acs.oprd.5b00417.
